# Assessing endometrial microbiota in endometriosis: culturomics and sequencing analysis of receptive-phase tissue

**DOI:** 10.1016/j.crmicr.2026.100593

**Published:** 2026-04-01

**Authors:** A. Canha-Gouveia, C.M. Tenorio, T. Rööp, S. Kõljalg, I. Smidt, E. Sepp, J. Štšepetova, M. Saare, S. Vela, I. Pérez-Prieto, E. Vargas, J. Mozas, A. Clavero, A. Sola-Leyva, A. Salumets, R. Mändar, S. Altmäe

**Affiliations:** a Department of Biochemistry and Molecular Biology, Faculty of Sciences, University of Granada, Granada, Spain; b Instituto de Investigación Biosanitaria ibs.GRANADA, Granada, Spain; c Department of Anatomy and Cell Biology, Faculty of Medicine, University of Cantabria, C/Cardenal Herrera Oria s/n 39011, Santander, Spain; d Department of Microbiology, Institute of Biomedicine and Translational Medicine- University of Tartu, Tartu, Estonia; e Laboratory of Clinical Microbiology and mycobacteriology, United Laboratories, Tartu University Hospital, Tartu, Estonia; f Celvia CC AS, Tartu, Estonia; g IVI-RMA Global Research Alliance, IVI Foundation, Instituto de Investigación Sanitaria La Fe (IIS La Fe), Valencia 46026, Spain; h Systems Biology Unit, Department of Experimental Biology, Faculty of Experimental Sciences, University of Jaen, Jaen, Spain; i U Reproducción, UGC Laboratorio clínico y UGC Obstetricia y Ginecología. HU Virgen de las Nieves, Granada, Spain; j Division of Obstetrics and Gynaecology, Department of Clinical Science, Intervention and Technology, Karolinska Institutet, Huddinge, Stockholm, Sweden; k Department of Gynaecology and Reproductive Medicine, Karolinska University Hospital, Huddinge, Stockholm, Sweden; l Department of Obstetrics and Gynaecology, Institute of Clinical Medicine, University of Tartu, Tartu, Estonia

**Keywords:** Culturomics, Endometriosis, Endometrium, Microbiome, Microbiota

## Abstract

Endometriosis has been associated with alterations in the reproductive tract microbiota, yet studies focusing on the endometrial microbiome remain inconsistent, partly due to the low-biomass nature of this niche and methodological heterogeneity. In this prospective pilot study, we investigated whether the receptive-phase endometrial microbiota differs between women with and without endometriosis using a combined culturomics and 16S rRNA gene sequencing approach. Endometrial samples were collected from 20 women undergoing fertility assessment, including 10 women with a previous diagnosis of endometriosis and 10 controls with male factor infertility. Biopsies were used for culturomics and Tao brush samples for sequencing, both obtained during the mid-secretory phase. Viable bacterial growth was recovered from 19 of 20 biopsies, yielding approximately 2500 colonies corresponding to 40 non-redundant species. Direct plating and aerobic pre-incubation shared around 45% of detected species, with pre-incubation enhancing species recovery but limiting quantitative comparisons. Bacterial load estimated from direct cultures showed wide inter-individual variability and no significant differences between groups, consistent with 16S rRNA gene sequencing results, which also revealed no clear differences in microbial diversity or overall community composition within this cohort. Only a limited overlap was observed between taxa detected by culture and sequencing, highlighting the complementarity and methodological biases of each approach. Overall, this study demonstrates the feasibility of recovering viable bacteria from receptive-phase endometrial samples and supports the combined use of culturomics and sequencing as complementary tools for studying microbiota in low-biomass uterine environments. These findings should, however, be interpreted considering the exploratory design and limited sample size.

## Highlight

• First study to explore live microbes in endometrial samples in women with endometriosis.
• Combined culturomics and 16S sequencing to study low-biomass endometrial microbiota.
• Viable bacteria were recovered from receptive-phase endometrial biopsies.
• Direct plating enables quantitative analysis, while pre-incubation expands species recovery.
• Culturomics and sequencing provide complementary but method-dependent microbial profiles.
• Women present individual microbial profiles with no consistent endometriosis specific endometrial microbiota signature.

## Graphical abstract

**Figure:**
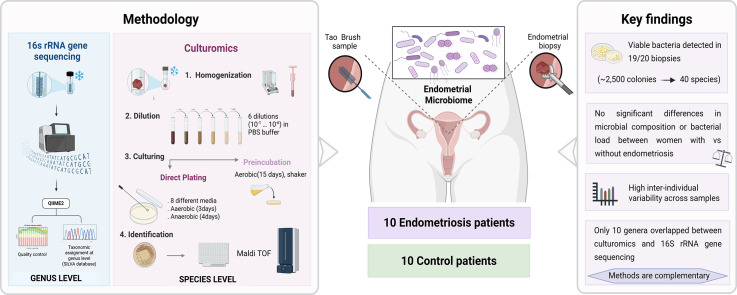

## 1 Introduction

Endometriosis is a chronic inflammatory disorder affecting approximately 10% of women of reproductive age (Bonavina and Taylor, 2022). It is characterised by the ectopic presence of endometrial-like tissue outside the uterine cavity and is associated with debilitating symptoms such as chronic pelvic pain, infertility, and impaired quality of life (Giudice, 2010). Despite its high prevalence, endometriosis is often associated with diagnostic delays, limited therapeutic efficacy, and an aetiology that remains poorly understood (Domínguez, 2018; Saunders and Horne, 2021; Soliman et al., 2017).

Recent evidence suggests that microbial factors may contribute to the pathogenesis of endometriosis by disrupting the uterine microenvironment and promoting lesion formation (Muraoka et al., 2024; Pérez-Prieto et al., 2024; Sobstyl et al., 2023). A reduction in *Lactobacillus*, an essential genus for maintaining microbial homeostasis in the female reproductive tract (Benner et al., 2018; Moreno et al., 2022), together with an overrepresentation of opportunistic or pathogenic bacteria, has been linked to dysbiosis and the development of endometriosis (Ata et al., 2019; Chadchan et al., 2019; Hernandes et al., 2020; Wei et al., 2020). More recently, studies have drawn attention to specific species such as *Fusobacterium nucleatum* as potential triggers of the disease (Muraoka et al., 2023). However, the mechanisms underlying these findings and their clinical relevance remain unclear. Moreover despite numerous microbiome studies in different cohorts, no consensus has been reached regarding the microbial factors potentially involved in endometriosis onset or progression (Garcia-Velasco et al., 2025; Graciano-España et al., 2025; Muraoka et al., 2024; Wei et al., 2020). This may partly reflect the limitations of the approaches most commonly used to characterise the microbiome, such as whole-metagenome and 16S rRNA gene sequencing, particularly in low-biomass tissues like the endometrium (Molina et al., 2020). These methods are prone to contamination, PCR bias, restricted taxonomic resolution and, importantly, cannot distinguish viable from non-viable microbes (Altmäe and Rienzi, 2021; Lagier et al., 2018; Molina et al., 2021; Sola-Leyva et al., 2021). This complicates both the biological interpretation of sequencing data and the potential clinical translation of microbial modulators, including antibiotics, prebiotics and probiotics, which act only on living bacteria (Altmäe and Rodríguez-Santisteban, 2025). Consequently, defining the active endometrial microbiota has become a priority to better understand and manage dysbiosis in endometriosis. This need has renewed interest in culture-based methods, which inherently target viable organisms and therefore provide a direct way to distinguish living from dead bacteria (Dewi Puspita et al., 2012; Lagier et al., 2015). Their use was historically limited by the belief that many species were unculturable, when in fact many are simply slow-growing, nutrient-dependent, or suppressed by more competitive strains, making the conventional agar-based techniques inadequate (Vanstokstraeten et al., 2023). Culturomics was developed to overcome these limitations by combining diverse culture conditions (e.g. multiple media, temperatures and atmospheric environments) with high-throughput identification such as MALDI-TOF mass spectrometry (Dewi Puspita et al., 2012; Lagier et al., 2015; Naud et al., 2020; Vanstokstraeten, et al., 2023). This novel approach has recently been applied to study the viable endometrial microbiome in infertile women, using different sample types such as catheter tip residues (Smolnikova 2019), endometrial fluid (Cariati et al., 2023) and biopsies (Vanstokstraeten et al., 2022; Vanstokstraeten, et al., 2023) and it was effective in demonstrating that the endometrium harbours viable microorganisms with profiles distinct from the vaginal microbiota (Cariati et al., 2023; Vanstokstraeten, et al., 2023). However, these studies varied substantially in culture conditions and analytical scope, making direct comparison difficult and limiting their interpretation as comprehensive culturomics surveys. In this context, the term “culturomics” is used here to describe a culturomics-based strategy aimed at recovering viable bacteria under a defined set of experimental conditions, rather than an exhaustive cultivation of the endometrial microbiota. Although these studies provide a proof-of-concept that culturomics is a feasible tool to characterise the viable endometrial microbiome despite being a low microbial biomass site, it has not yet been applied to women with endometriosis to determine whether the disease is associated with distinct viable endometrial bacteria.

In this exploratory pilot study, we assessed the feasibility and added value of combining culture-based and sequencing approaches in endometrial samples from a small cohort of women with and without endometriosis. Our objectives were to characterise the viable endometrial microbiota, to evaluate the added value of pre-incubation for species recovery, to quantify bacterial load across groups, and to compare these results with conventional microbiome profiling by 16S rRNA gene sequencing in matched samples.

## 2 Materials and methods

### 2.1 Study population and ethical approval

This cross-sectional pilot study included endometrial samples from 20 Caucasian women undergoing fertility assessment, at the Reproduction Unit, Virgen de las Nieves University Hospital (Granada, Spain), between March 2019 and April 2022. Written informed consent was obtained from all participants prior to inclusion. All participants had experienced at least one year of unprotected intercourse without conception and had completed a comprehensive infertility workup before enrolment. Ten women were diagnosed with endometriosis and were not receiving any treatment for the condition. The control group consisted of ten women without diagnosed gynaecological abnormalities, whose inability to conceive was attributed to the male factor. Exclusion criteria included age ≥43 years, hormonal treatment at the time of recruitment, presence of gynaecological tumours, systemic diseases, or other gynaecological conditions unrelated to endometriosis. The study was approved by the Ethics Committee of the Junta de Andalucía (approval number CEIM/CEI 0463-M1–18r) and conducted in accordance with the Declaration of Helsinki and Spanish Law 14/2007 of July 3 on Biomedical Research.

### 2.2 Sample collection and processing

Endometrial sampling was scheduled individually according to each participant’s menstrual cycle to ensure collection during the mid-secretory phase. The urinary LH surge was detected using commercial home tests (ClearBlue®, Swiss Precision Diagnostics GmbH, Geneva, Switzerland), and sampling was arranged 7–9 days after the reported peak (LH+7 to LH+9).

**Fig. 1:**
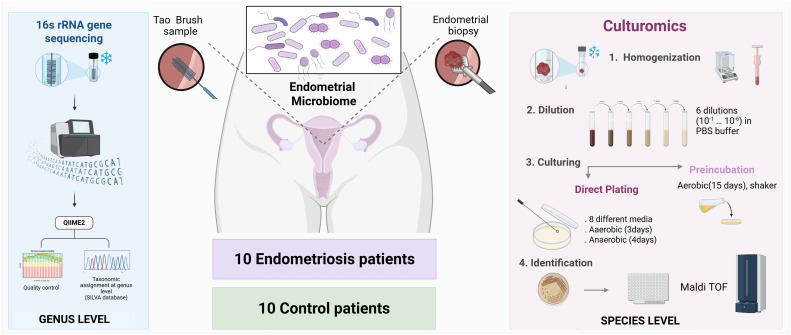
Workflow overview of microbiota analysis in endometrial samples from women with endometriosis and non-endometriosis controls. Two types of endometrial samples were collected: Tao brush samples for 16S rRNA gene sequencing and biopsies for culturomics analyses. 16S rRNA sequencing pipeline included DNA extraction, amplification of the V4 hypervariable region, and taxonomic classification—allowing bacterial identification at the genus level. Culturomics workflow comprised of (1) sample homogenization and serial dilution; (2) bacterial cultivation through two strategies—direct plating and aerobic preincubation; (3) morphological selection of distinct colonies; and (4) identification of isolated strains by MALDI-TOF mass spectrometry. This approach enabled bacterial identification down to the species level and the recovery of viable microorganisms for further study.

At the scheduled visit, participants were placed in the lithotomy position. A speculum was inserted without lubricant to visualise the cervix and allow transcervical introduction of the sampling devices while minimising contamination. Two samples were then collected sequentially using the devices most reported in the literature for each microbiome analysis approach: an endometrial brushing with a Tao Brush IUMC (Cook Medical, Madrid, Spain) for 16S rRNA gene sequencing, and an endometrial biopsy with a Pipelle de Cornier (Gynétics, Belgium) for culturomics analysis (Fig. 1).

First, the Tao Brush was inserted transcervically with its protective sheath. Once inside the uterine cavity, the sheath was retracted and the brush gently rotated to collect endometrial cells. The sheath was then re-covered and the brush tip was aseptically detached using sterile, single-use cutting instruments, and transferred into an eNAT® 606C transport tube (Copan Diagnostics, Italy) designed for nucleic acid preservation, and stored at −80 °C. Afterwards, an endometrial biopsy (1–3 mm³) was obtained using a Pipelle de Cornier (Gynétics, Belgium) for culturomics analysis. The tissue was divided into two fractions: one snap-frozen in cryotubes and stored at −80 °C, and the other fixed in 10% buffered formalin for histological dating. Histological dating was performed on haematoxylin and eosin–stained sections according to the Noyes criteria, independently by two experienced pathologists. Chronic endometritis was assessed by CD138/MUM1 immunohistochemistry, and no samples showed evidence of plasma cell infiltration.

Environmental and procedural controls were collected in parallel during the sampling period and processed alongside clinical samples for 16S rRNA gene sequencing, allowing assessment of potential background contamination in this low-biomass setting. Swabs of gloves, Tao Brush cutter tools, blank Copan transport tubes, and air samples from the procedure room were placed into Copan eNAT® tubes and processed together with sequencing samples. As the eNAT® medium does not support bacterial viability, these controls could not be subjected to culturomics but were analysed by 16S rRNA sequencing. Commensal bacteria detected in control samples were interpreted as part of the expected microbial background of the upper reproductive tract. For sequencing data, taxa detected in environmental and procedural controls were addressed through in silico decontamination using the microDecon approach, as described below.

### 2.3 Culture-Based microbiota analysis using culturomics

Frozen endometrial biopsies were thawed, weighed, and homogenised in 200 μL sterile phosphate-buffered saline (PBS; Sigma) using tissue grinders. Two culturomics strategies were applied. In the direct plating approach, serial dilutions (10⁻¹ to 10⁻⁶) were plated (100 μL) onto eight different solid media (Supplementary Table S1) and incubated under aerobic, anaerobic, and microaerobic conditions. In the pre-incubation approach, 500 μL of the 10⁻¹ dilution was inoculated into BD BACTEC™ Plus Aerobic vials and incubated at 37 °C for 14 days, after which 100 μL of broth was plated on the same media panel. All conditions were tested in duplicate.

Colony morphotypes were isolated and subcultured to obtain pure colonies. Identification was performed by MALDI-TOF MS (Bruker Biotyper Sirius), with isolates spotted onto steel target plates, treated with 70% formic acid and matrix solution, and analysed using FlexControl v3.4 and MBT Compass v4.1 software. Only identifications with a score ≥2.0 were considered reliable at the species level. The taxonomic classification at the phylum, class, order, family, genus and species levels and the Gram status was assigned according to the List of Prokaryotic names with Standing in Nomenclature (LPSN) (Meier-Kolthoff et al., 2022; Parte et al., 2020) the NCBI Taxonomy database (Schoch et al., 2020), and the phylogenetic framework proposed (Oren and Garrity, 2021). When necessary, interactive visualisation and phylogenetic positioning were cross-checked using iTOL v6 (Letunic and Bork, 2024). All taxonomic assignments were verified as of August 2025. The frequency of detection for each species was summarised as both the absolute number of patients with positive culture (1–10) and the percentage of women in whom the bacterium was isolated. Endometriosis and control groups were represented separately, and results are displayed for both direct plating and pre-incubation culture strategies. For transparency, isolate-level metadata were compiled for all culturable taxa and are provided in Supplementary Files A and B. These files include, for each patient, the culture medium and atmospheric condition supporting growth, MALDI-TOF identification scores, colony counts (CFU/g tissue), and oxygen-tolerance classification. Supplementary File A corresponds to control samples and Supplementary File B to endometriosis cases.

Quantification of bacterial load was restricted to colonies obtained through direct plating. Colony-forming units per gram (CFU/g) were calculated by multiplying colony counts by the dilution factor and normalising by tissue weight. The detection limit was established at 3.0 log CFU/g. Quantification from pre-incubated samples was excluded due to the risk of artificial overgrowth.

### 2.4 16S rRNA gene sequencing

DNA was extracted from Tao Brush samples using the Qiagen QIAamp UCP Pathogen Mini Kit with Pathogen Lysis Tube S, following the manufacturer’s protocol. DNA concentration and purity were assessed using NanoDrop ND-1000 (Thermo Fisher Scientific, Waltham, MA, USA) and Qubit 4 fluorometer (Thermo Fisher Scientific). Negative controls comprised sample collection controls specific to each tissue source, as well as controls for DNA extraction reagents, library preparation, and sequencing procedures. Positive controls consisted of the ZymoBIOMICS™ microbial community standard (Zymo Research, Irvine, CA, USA).

Amplification of the V4 hypervariable region of the 16S rRNA gene was performed using primers 515F −5′-GTGYCAGCMGCCGCGGTAA-3′ and 806R-5′ -ACTACNVGGGTWTCTAAT-3′. PCR reactions (25 μL) included 5 μL of each primer (1 μM), 12.5 μL of 2 × KAPA HiFi HotStart ReadyMix, and 2.5 μL of DNA (10 ng). Cycling conditions were: 95 °C for 3 min; 35 cycles of 95 °C for 30 s, 55 °C for 30 s, and 72 °C for 30 s; followed by a final extension at 72 °C for 5 min.

Amplicons (∼380 bp) were verified on 2% agarose gels and purified using AMPure XP beads. Indexed libraries were prepared following the Illumina 16S Metagenomic Sequencing Library Preparation protocol. Library quality was assessed via Bioanalyzer using a High Sensitivity kit. Sequencing was performed on the Illumina MiSeq platform (2 × 300 bp) using the MiSeq Reagent Kit v3.

Demultiplexed reads were processed in QIIME2 v2023.9. Quality filtering and denoising were conducted using DADA2. Taxonomic assignment was performed using the SILVA v138.1 database. Rarefaction curves were generated to assess sequencing depth; samples with insufficient reads were excluded. Decontamination was performed *in silico* using the microDecon R package, based on taxa found in environmental controls.

### 2.5 Data analysis

All analyses were conducted using SPSS v27.0.1.0 and R v4.4.2 (RStudio 2024.10.31). For culturomics, only bacterial taxa detected in ≥20% of samples per group were included in the comparative analyses. Quantitative data expressed as log10 CFU/g were compared between the women with endometriosis and controls using the non-parametric Wilcoxon rank-sum test. The number of positive samples (n > 0), median values, and maximum counts were summarised for each group. A two-sided p-value < 0.05 was considered statistically significant, without adjustment for multiple comparisons given the exploratory nature of this pilot study.

For microbiome analyses based on 16S rRNA gene sequencing, data were processed and visualised using the phyloseq, vegan, microviz and ggplot2 packages. To further assess the robustness of the sequencing results, analyses were performed across multiple taxonomic levels, including phylum, class, order, family and genus. Alpha diversity was estimated with the Shannon diversity index and richness (number of genera), calculated with the “diversity” and “specnumber” functions from the vegan package. Group comparisons were performed by Analysis of Covariance (ANCOVA), adjusting for age, BMI and antibiotic exposure. Beta diversity was evaluated using Bray-Curtis dissimilarity and visualised by both principal coordinates analysis (PCoA) and non-metric multidimensional scaling (NMDS). Group differences were tested by permutational analysis of variance (PERMANOVA) using the “adonis2” function. Differential abundance was evaluated with Analysis of Compositions of Microbiomes with Bias Correction (ANCOM-BC) implemented in the ancombc2 package(Lin and Peddada, 2020). This method estimates absolute abundances within a linear regression framework while adjusting for BMI, age and antibiotic exposure. Prior to differential abundance analysis, a minimum relative abundance threshold of 0.1% was applied to exclude low-frequency taxa and reduce noise (Cao et al., 2020; Nikodemova et al., 2023). For sequencing analyses, statistical significance was set at q < 0.05 after Benjamini–Hochberg false discovery rate (FDR) correction. Schematic figures were created with BioRender and using the Venny web tool (Venny 2.1.0: Venn Diagram Plotter)(Oliveros, 2015). Reporting of this study was guided, where applicable, by the STORMS (Strengthening The Organization and Reporting of Microbiome Studies) recommendations, taking into account the exploratory nature of this pilot study and the constraints inherent to low-biomass endometrial samples.

## 3 Results

### 3.1 Study group characteristics

The mean age and BMI of women with endometriosis were 33.4 ± 4.03 years and 22.78 ± 4.04 kg/m², respectively, while those of the control group were 34.3 ± 3.89 years and 24.74 ± 4.00 kg/m². No statistically significant differences were observed between the groups (age, p = 0.62; BMI, p = 0.29). Individual data are provided in Supplementary Table S2, and detailed clinical characteristics of the endometriosis group are summarised in Supplementary Table S3.

As part of the routine infertility evaluation, histology assessment of the endometrial tissue for menstrual phase and screening for chronic endometritis were performed, together with microbiological testing for commensal and pathogenic genital microorganisms. Analyses were conducted independently by the hospital’s diagnostic laboratories using culture and PCR-based methods for *Chlamydia trachomatis, Neisseria gonorrhoeae, Trichomonas vaginalis, Ureaplasma parvum, Ureaplasma urealyticum, Mycoplasma genitalium,* and *Mycoplasma hominis* (Supplementary Table S4). All patients were confirmed to be in the mid-secretory phase at the time of biopsy. Commensal organisms such as *Lactobacillus* spp*., Gardnerella vaginalis, and Enterococcus faecalis* were reported in several controls, while one endometriosis patient tested positive for *N. gonorrhoeae* by *PCR*.

### 3.2 Microbiota assessment by culturomics

All culture-based findings were interpreted as the recovery of viable bacteria under the specific experimental conditions applied, rather than as a direct representation of the *in vivo* endometrial community. Of the 20 biopsies analysed, 19 (95%) yielded viable bacterial growth, resulting in ∼2500 colonies corresponding to 40 distinct species. In total, 22 genera were identified across 15 families, 10 orders, 5 classes, and 3 phyla, with Gram-positive bacteria predominating (92.5%) (Supplementary Table S5).

**Fig. 2:**
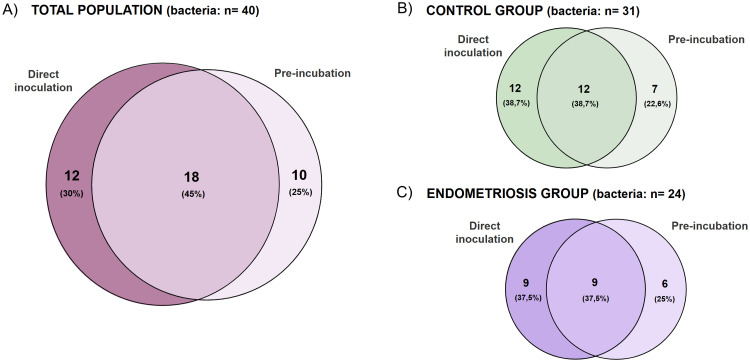
Venn diagrams comparing bacterial species isolated by direct inoculation and pre-incubation. The A panel shows the overall distribution in the total study population, with 12 species (30%) detected only by direct inoculation, 10 (25%) only after pre-incubation, and 18 (45%) by both strategies. The right panels illustrate the overlap within each clinical group: in controls (panel B), 12 species (38.7%) were recovered exclusively by direct inoculation, 7 (22.6%) only after pre-incubation, and 12 (38.7%) by both methods (total n = 31), and in endometriosis (purple, bottom right), 9 species (37.5%) were isolated only by direct inoculation, 6 (25%) only after pre-incubation, and 9 (37.5%) by both methods (total n = 24). Percentages indicate the proportion relative to the total number of species in each group, and diagrams were generated using the Venny web tool (Venny 2.1.0: Venn Diagram Plotter) (Oliveros, 2007–2015).

In the comparison of culturomics strategies (Fig. 2), direct inoculation and pre-incubation yielded partly overlapping but distinct bacterial profiles. In the overall study population, 30% of the species were isolated exclusively by direct inoculation, 25% only after pre-incubation, and 45% by both methods. When stratified by clinical group, control samples yielded a total of 31 species, with 38.7% detected only by direct inoculation, 22.6% only after pre-incubation, and 38.7% by both approaches. In endometriosis samples (n = 24 species), 37.5% were isolated exclusively by direct inoculation, 25% only after pre-incubation, and 37.5% by both methods. These findings indicate that the pre-incubation and direct inoculation are complementary strategies for recovering viable endometrial bacteria.

**Fig. 3:**
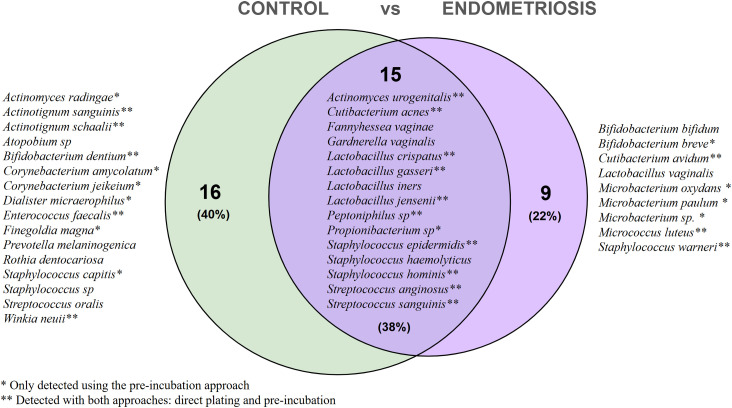
**Comparative distribution of bacterial species identified by culturomics in endometrial samples from control and endometriosis groups.** Species were isolated using both direct inoculation and pre-incubation strategies. Overall, 15 species were shared between groups, 16 were unique to controls, and 9 were specific to endometriosis. Species labels without an asterisk correspond to isolates recovered exclusively by direct inoculation; * indicates species detected only after pre-incubation; ** indicates species recovered by both direct inoculation and pre-incubation. Comparative distribution of species between groups was visualised with a Venn diagram generated using the Venny web tool (Venny 2.1.0: Venn Diagram Plotter) (Oliveros, 2007–2015). Percentages indicate the proportion of species relative to the total number identified.

When comparing study groups with both culture methods, 15 species were shared between the controls and endometriosis groups (Fig. 3).

**Fig. 4:**
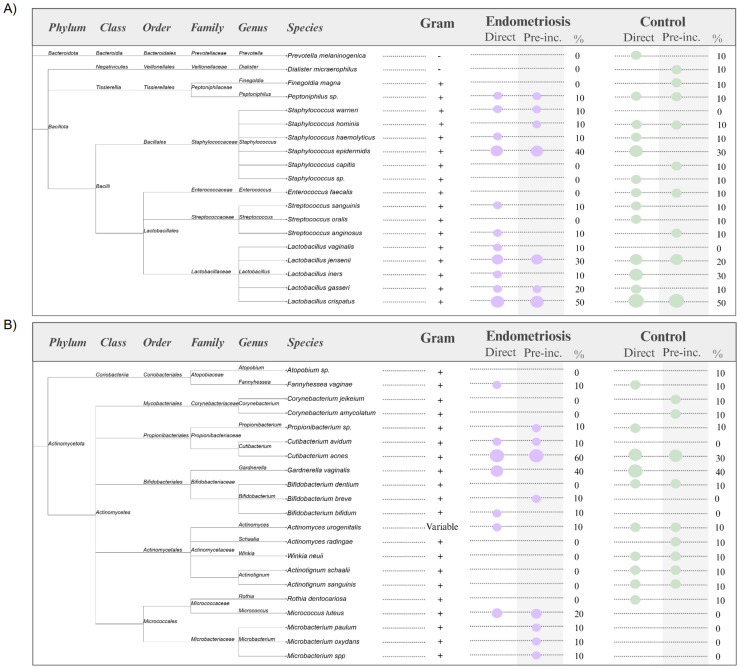
**Taxonomic classification and frequency of viable endometrial bacteria detected by culturomics in women with and without endometriosis.** Panel A shows taxa belonging to the phylum *Bacteroidota* (n = 1) and *Bacillota* (n = 18), while panel B depicts taxa from the phylum *Actinomycetota* (n = 21). Bacterial isolates were classified according to their phylum, class, order, family, genus and species, and annotated as Gram-positive or Gram-negative following the LPSN, NCBI Taxonomy and recent phylogenetic revisions (Meier-Kolthoff et al., 2022; Oren and Garrity, 2021; Parte et al., 2020; Schoch et al., 2020). Circle size represents the number of patients in which each species was identified (smallest = 1 patient, largest = 10 patients). Columns display detection rates in endometriosis and control groups, both after direct plating and following pre-incubation. The percentage of positive patients is indicated in the adjacent column. Figure created with BioRender (Altmäe, S. 2025; https://BioRender.com/wlzfntm).

Nine species were detected exclusively in endometriosis. Four were recovered only after pre-incubation (*Microbacterium oxydans, Microbacterium paulum, Microbacterium* sp.*, Bifidobacterium breve*), while others were obtained by direct culture (*Bifidobacterium bifidum, Lactobacillus vaginalis*) or by both methods (*Cutibacterium avidum, Micrococcus luteus, Staphylococcus warneri*). In contrast, 16 species were unique to controls, including *Actinotignum sanguinis, Bifidobacterium dentium,* and *Enterococcus faecalis. Lactobacillus crispatus* was the most prevalent taxon, present in 50% of participants in both groups (Fig. 4A). *Cutibacterium acnes* was the second most frequent, isolated from six endometriosis patients and three controls. Gram-negative species such as *Dialister micraerophilus* and *Prevotella melaninogenica* were detected only in controls, while *Gardnerella vaginalis* (Gram-variable) was found in four individuals per group (Fig. 4B).

**Fig. 5:**
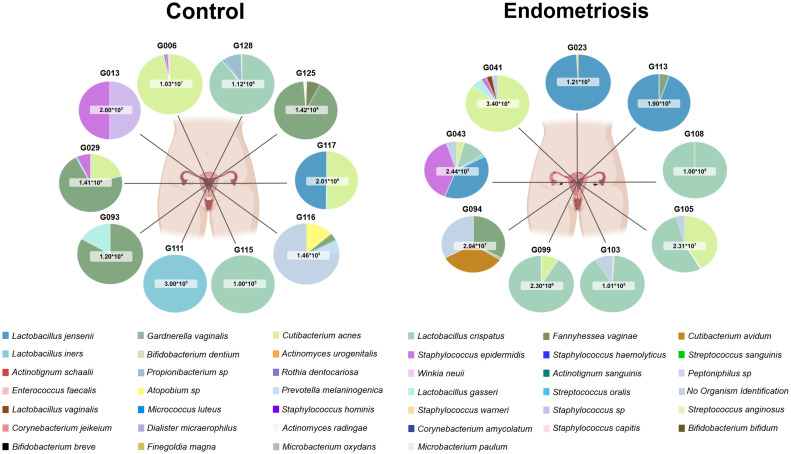
**Patient-specific bacterial composition.** The number of CFU/g is indicated per sample. Each colour represents a bacterial species. The legend lists taxa from highest to lowest relative abundance across samples. Created in BioRender. Altmäe, S. (2025)https://BioRender.com/bqgrexd.

From a metabolic perspective, isolates comprised facultative anaerobes (47.5%), strict anaerobes (35%), aerobes (12.5%) and aerotolerant anaerobes (5%), with no significant group differences (Supplementary Table S5, Supplementary Files A and B). Overall, Gram-positive bacteria predominated (Supplementary Table S6). In addition, at the individual level, most species (25 of 40) were recovered from a single participant (G125), reflecting high inter-individual variability in the composition of endometrial bacterial isolates (Supplementary Tables S7–S8). These patterns are illustrated in patient-specific colony-forming unit counts (CFU/g) (Fig. 5, Figure S1 and Figure S2).

**Fig. 6:**
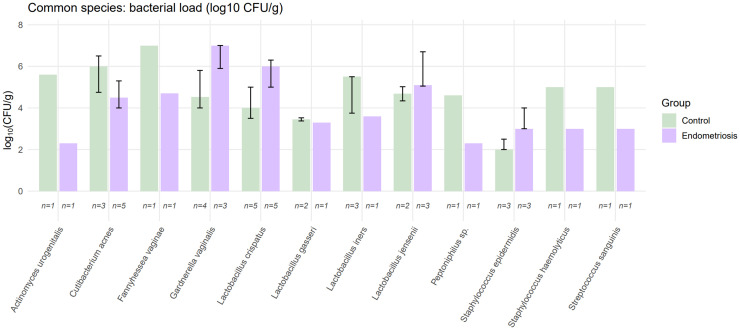
**Bacterial load (log₁₀ CFU/g) of common species isolated from endometrial samples in control and endometriosis groups.** Boxplots show the distribution of colony-forming units per gram for each species, and labels below each box indicate the number of positive samples (n). No significant differences were observed between groups.

Bacterial load, expressed as log₁₀ CFU/g, did not differ significantly between the endometriosis and control groups for any of the 12 shared species analysed by direct inoculation (Wilcoxon rank-sum tests, all p > 0.05; Fig. 6, Supplementary Tables S9–S10).

Among these taxa, *Cutibacterium acnes, Gardnerella vaginalis, Lactobacillus crispatus,* L. *gasseri,* L. *iners,* L. *jensenii* and *Staphylococcus epidermidis* were detected in ≥20% of participants. For five of these species, the median CFU/g was zero in both groups, reflecting their presence at low relative abundance despite being frequently detected (Fig. 6). Although some species, such as *Cutibacterium acnes* and *Lactobacillus inners*, showed numerically higher median loads in controls, the overall distributions overlapped extensively, with no statistically significant differences between the study groups.

### 3.4 16S rRNA gene sequencing

**Fig. 7:**
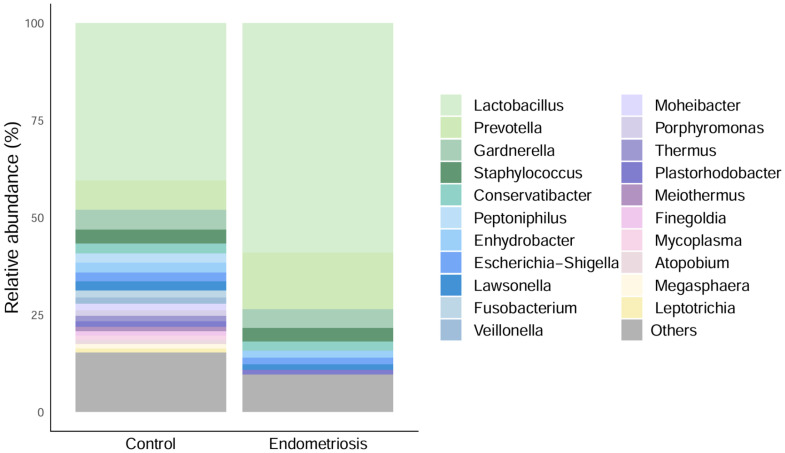
**Mean relative abundances of the genera identified in controls and women with endometriosis with 16S rRNA gene sequencing.** Only genera with a relative abundance of >0.1% were included. To facilitate visualization, genera with abundances between 0.1% and 1% were grouped as “Others”.

Sequencing analyses confirmed marked inter-individual variability in endometrial microbial composition. Shannon diversity was significantly lower in women with endometriosis compared with controls (ANCOVA adjusted for age and BMI, p = 0.008), whereas richness did not differ significantly (p = 0.109) (Figure S3A). Beta diversity analysis (PCoA based on Bray–Curtis’s dissimilarity) showed no significant differences in overall community composition between groups (PERMANOVA, R² = 0.094, p = 0.072; Figure S3B). To further assess the robustness of the sequencing results, we performed an expanded re-analysis across multiple taxonomic levels (phylum, class, order, family and genus). Across these taxonomic resolutions, alpha diversity metrics did not show consistent significant differences between women with endometriosis and controls (Figure S4A,B,E,F,I,J,M,N,Q,R). Although a trend towards lower Shannon diversity in endometriosis was observed at some taxonomic levels (e.g. class and family; p ≈ 0.09–0.10), these differences were not statistically significant after adjustment for age, BMI and antibiotic exposure. Notably, some associations with covariates were detected, including BMI and antibiotic exposure at specific taxonomic levels, but these effects were not consistent across analyses. Similarly, beta-diversity analyses based on Bray–Curtis dissimilarity did not reveal any clear group separation, either by PCoA or by NMDS (Figure S4C,D,G,H,K,L,O,P,S,T). PERMANOVA analyses confirmed the absence of significant differences in overall community composition between groups across taxonomic levels (all p > 0.05; R² < 0.1). Differential abundance testing using ANCOM-BC, adjusted for BMI, age and antibiotic exposure, identified some nominal associations at specific taxonomic levels; however, none remained significant after false discovery rate correction (Figure S5). Relative abundance plots across taxonomic levels showed that microbial communities in both groups were dominated by similar major taxa, without evident structural shifts between controls and endometriosis samples (Figure S5A–D). At the genus level, *Lactobacillus* was dominant in both groups, with a mean relative abundance of 40.5% in controls and 59.1% in endometriosis (Fig. 7).

Other prevalent genera included *Prevotella, Gardnerella, Staphylococcus, Conservatibacter, Enhydrobacter, Escherichia–Shigella, Lawsonella* and *Plastorhodobacter*. No significant group-specific differences in overall taxonomic composition were detected. Overall, these profiles were highly heterogeneous across individuals and did not support a consistent disease-specific compositional pattern.

Several low-abundance taxa detected in patient samples were also present in the negative controls and were therefore removed *in silico* using the microDecon algorithm. A detailed list of the taxa identified in environmental controls is provided in Supplementary Table S11.

### 3.5 Complementarity of culturomics and 16S rRNA gene sequencing

**Fig. 8:**
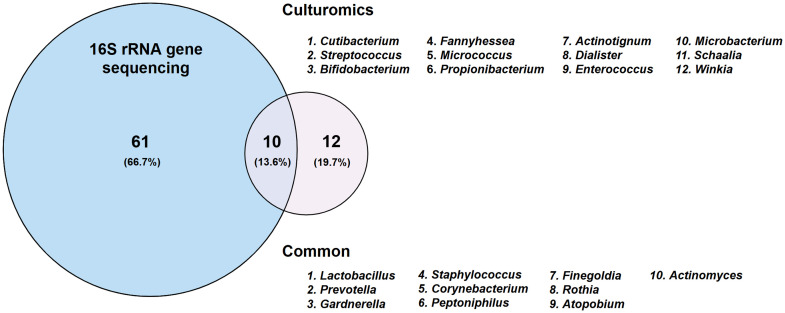
**Overlap between bacterial taxa identified by culturomics and 16S rRNA gene sequencing in endometrial samples from the 20 women included in the study.** Comparative distribution of taxa detected by both approaches was visualised using Venn diagrams generated with the Venny web tool (Venny 2.1.0: Venn Diagram Plotter) (Oliveros, 2007–2015). 16S rRNA gene sequencing detected 61 taxa (66.7%) exclusively, culturomics identified 12 taxa (19.7%) exclusively, and 10 taxa (13.6%) were shared by both methods. Percentages refer to the proportion of taxa relative to the total number of species identified.

Direct comparison of paired samples highlighted the complementarity of both approaches. Culturomics identified 12 genera absent from 16S rRNA profiles, such as *Cutibacterium, Enterococcus,* and *Micrococcus*, while 16S rRNA gene sequencing detected 61 genera not recovered by culturomics, including *Blautia, Fusobacterium, and Mycoplasma* (Supplementary Table S12). Only 10 genera were shared across the methods, including *Lactobacillus, Gardnerella, and Staphylococcus* (Fig. 8).

**Fig. 9:**
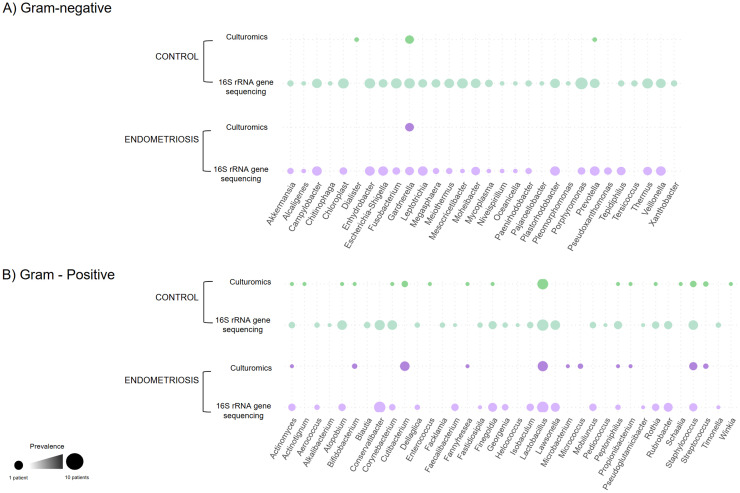
**Presence of Gram-negative (panel A) and Gram-positive (panel B) bacterial genera identified in receptive-phase endometrial samples by culturomics and 16S rRNA gene sequencing.** Dots indicate presence in at least one individual within that group. The figure illustrates the distinct taxonomic profiles recovered by each method, with partial overlap and method-specific detections.

Methodological biases were evident: culturomics favoured recovery of Gram-positive genera, while 16S rRNA gene sequencing captured a broader range of Gram-negative and fastidious taxa (Fig. 9).

**Fig. 10:**
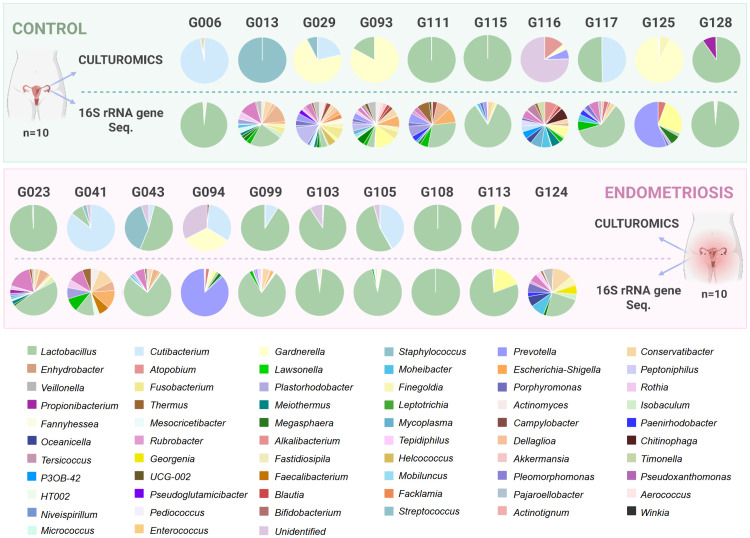
**Microbial profiles at genus level, obtained using 16S rRNA gene sequencing and culture-based (culturomics) approaches, in receptive-phase endometrial samples from women diagnosed with endometriosis and controls.** Each colour represents a bacterial genus. The legend lists taxa from highest to lowest relative abundance across samples. Created in BioRender. Altmäe, S. (2025)https://BioRender.com/qjuvqhs.

At the individual level, microbial profiles differed markedly among participants regardless of the group or technique (Fig. 10). One sample (G124) yielded no culturable bacteria, although 16S sequencing revealed a wide range of genera. Differences between culture-based and sequencing-based profiles likely reflect methodological biases intrinsic to each approach, as well as the use of different sample types, rather than biological discordance alone.

## 4 Discussion

This pilot study aimed to characterise the endometrial microbiota in women with and without endometriosis using complementary approaches, culturomics and 16S rRNA gene sequencing. We detected a variety of bacterial taxa across samples, but no consistent disease-specific microbial pattern was identified. Both techniques revealed a predominance of *Lactobacillus* spp*.* and a high degree of inter-individual variability, with no statistically significant differences in microbial diversity or bacterial load between the groups. Moreover, expanded 16S rRNA gene re-analysis across multiple taxonomic levels, including alternative ordination approaches and covariate-adjusted differential abundance testing, converged on the same overall interpretation. Together, these analyses did not identify robust or consistent microbiome differences between women with endometriosis and controls. Therefore, these results do not support the presence of a consistent disease-specific microbial signature in the endometrium within our cohort. The hypothesis that endometriosis may be influenced by bacterial contamination or specific dysbiosis has gained attention in the recent years, particularly regarding the potential role of *Fusobacterium nucleatum* (Muraoka et al., 2023; Muraoka et al., 2024). In our cohort*, F. nucleatum* was detected by 16S rRNA sequencing in a few individuals, but not by culturomics, and no reproducible pattern was observed. The absence of *F. nucleatum* in culture-based results is not unexpected, as this species is notoriously difficult to cultivate and requires highly specific anaerobic conditions and enriched media. Its inconsistent detection across studies may therefore reflect methodological limitations rather than true absence. This highlights a critical challenge for microbiome research in low-biomass niches: taxa of potential interest may be systematically underrepresented depending on the analytical strategy employed. Future culturomics protocols should consider the targeted inclusion of selective media and optimised anaerobic systems to improve the recovery of such fastidious organisms.

Nine species were detected exclusively in endometriosis samples. Although most were isolated from single individuals, their presence highlights the potential for idiosyncratic microbial signatures in a subset of women rather than a reproducible disease-wide pattern. Such findings emphasise the high interpersonal variability of the endometrial microbiota and the difficulty of distinguishing true disease associations from stochastic colonisation events.

At sequencing level, we identified a broad range of bacterial genera, including many previously reported in studies of endometrial microbiome in endometriosis (Bednarska-Czerwińska et al., 2022; Canha-Gouveia et al., 2023; Moreno et al., 2016). No clear group-specific associations were detected. It is important to acknowledge that we used different types of samples for the two methodologies (biopsies for culturomics and Tao brush samples for sequencing), which may partly explain the discrepancies between the taxa recovered by each method. In one case (G124), no bacterial growth was obtained, whereas sequencing revealed diverse taxa, which could reflect low bacterial abundance below the threshold for culturing. Differences between culture-based and sequencing-based profiles may also reflect inherent methodological biases associated with amplicon-based approaches. In low-biomass tissues such as the endometrium, 16S rRNA gene sequencing is particularly susceptible to several sources of bias that may influence the interpretation of microbial profiles. These include differential DNA extraction efficiency across taxa, PCR amplification biases related to primer selection, stochastic amplification of low-abundance templates, and the difficulty of distinguishing true biological signals from background contamination. These methodological challenges have been widely discussed in previous studies of the endometrial microbiome and highlight the need for careful interpretation of sequencing-based results in low-biomass environments (Altmäe and Rienzi, 2021; Lagier et al., 2018; Molina et al., 2021; Sola-Leyva et al., 2021).

Despite the detection of multiple taxa, only a few appeared functionally active, as demonstrated by viable bacterial growth. Quantitative culturomics revealed that only *Lactobacillus crispatus* and *Cutibacterium acnes* consistently reached measurable CFU/g values and median CFU/g values above zero, although no significant differences were observed between the groups. This suggests that these species are among the most resilient colonisers of the endometrium. The recurrent detection of *Lactobacillus crispatus* aligns with its recognised role as a stabilising and protective member of the reproductive tract microbiota (Decout et al., 2024), whereas the frequent isolation of *Cutibacterium acnes* is consistent with its ability to persist in low-biomass niches, though its clinical relevance remains debated (Chen et al., 2023). The absolute loads observed (e.g. *G. vaginalis* 1.3 × 10⁸ CFU/g, *E. faecalis*3 × 10⁴ CFU/g in individual cases) indicate that viable bacteria are present in the endometrium, albeit at relatively low abundance. Nevertheless, even small amounts of viable bacteria may have biological relevance in the endometrium, as microbial components can activate innate immune receptors such as Toll-like receptors(Benner et al., 2018). Altered immune cell populations and cytokine responses have been described in the eutopic endometrium of women with endometriosis (Vallvé-Juanico et al., 2019), raising the possibility that low-level microbial signals could contribute to these changes. This concept is consistent with recent reports suggesting that uterine microbiota, although of low biomass, may influence endometrial receptivity and reproductive outcomes(Benner et al., 2018; Wessels et al., 2021).

This pronounced interpersonal heterogeneity aligns with the previous reports suggesting that the endometrial microbiota is strongly influenced by host-specific factors, including immune environment, hormonal status, and possibly subtle contamination from the lower genital tract (Krog et al., 2022; Toson et al., 2022). In this context, the search for a “universal” microbial signature of endometriosis may be misguided. Instead, disease-relevant effects could arise from functional interactions between individual-specific microbiota and the endometrium, such as modulation of local immunity or inflammatory signalling, rather than from the presence of specific taxa alone.

**Fig. 11:**
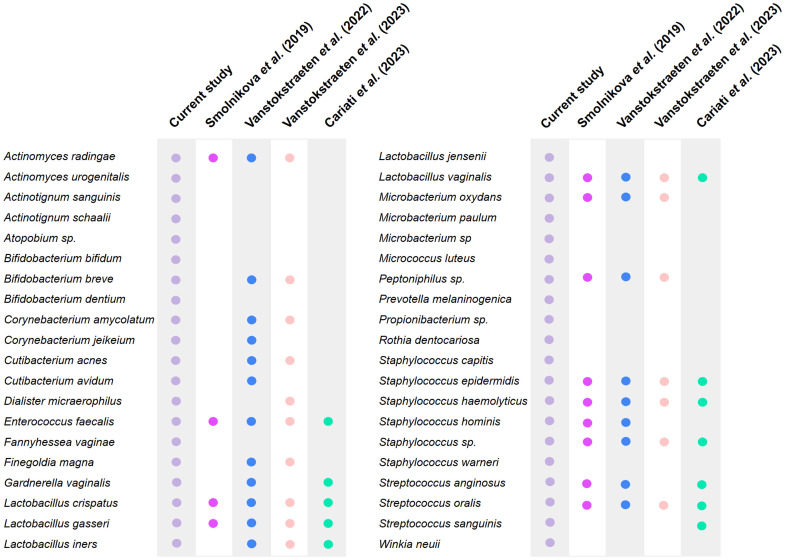
**Presence or absence of bacterial species isolated in our study (Canha-Gouveia et al.) compared with four previously published studies that also applied culturomics to the endometrium (Candelli et al., 2021; Cortez-Gonzalez et al., 2019; Sharma et al., 2019; Vitale et al., 2023).** Each row represents a bacterial species, and each column corresponds to one study. Coloured bubbles indicate species reported in the respective study, whereas the absence of a bubble denotes that the species was not identified.

Compared with previous culturomics studies (Cariati et al., 2023; Lagier et al., 2018; Vanstokstraeten et al., 2023; Vanstokstraeten et al., 2022, 2023), our dataset provides new insight into the viable microbiota of this low-biomass niche (Fig. 11).

Differences across studies may reflect methodological aspects such as sample preservation, as frozen biopsies were used for culture-based analyses, which may preferentially favour thermo- and aerotolerant organisms. Nevertheless, the recovery of viable *Lactobacillus, Gardnerella, and Enterococcus* spp. indicates that meaningful culturomics characterisation remains possible from stored specimens. In addition, despite careful sampling procedures, the possibility that some cultured taxa represent procedural or environmental carry-over cannot be entirely excluded in this low-biomass setting.

We also compared two culture strategies. The pre-incubation strategy was used to expand detectable diversity under controlled laboratory conditions and should not be interpreted as reflecting relative abundances or ecological dominance *in vivo*.

While direct inoculation and pre-incubation yielded overlapping species (18 shared), pre-incubation enabled the recovery of 10 additional taxa, whereas 12 were uniquely captured by direct plating. Pre-incubation steps have been previously described in culturomics workflows as enrichment strategies that facilitate the recovery of slow-growing or metabolically inactive bacteria (Dewi Puspita et al., 2012; Naud et al., 2020). In low-biomass environments such as the endometrium, this step may allow the enrichment of bacteria present at very low abundance, thereby increasing the probability of detection under laboratory conditions. The recovery of several *Microbacterium* species only after pre-incubation in our setting illustrates another methodological challenge. These organisms likely represent low-abundance or environmentally derived taxa whose detection depends on laboratory enrichment. Their growth under pre-incubation conditions may not necessarily reflect their *in vivo* relevance, underscoring the importance of interpreting culture-based detections considering potential artefacts introduced by enrichment strategies. However, since pre-incubation can artificially enrich microbial growth, only direct inoculation was used for quantitative comparisons. Importantly, the complementary strengths of culturomics and sequencing argue for an integrated strategy in the future research. Sequencing provides broad detection of low-abundance and fastidious taxa but cannot distinguish viable from dead bacteria. Culturomics, in contrast, demonstrates which organisms are metabolically active and quantify their load, but is inherently limited by culture conditions. Combining both approaches offers a more realistic picture of the endometrial ecosystem, allowing researchers to move beyond taxonomic lists towards hypotheses on microbial function and host–microbe interactions. The limitations of this study include a relatively small sample size, single-centre design, the employment of different sampling devices for culturomics and sequencing, and the use of snap-frozen tissue for culturomics, which may reduce the viability of certain fastidious anaerobes. However, previous studies have demonstrated that prompt cryopreservation preserves overall microbial diversity (Fouhy et al., 2015; Sepp et al., 2013), and in our study, both facultative and strict anaerobes were successfully recovered from frozen endometrial tissue. Future studies including larger patient cohorts will be essential to increase statistical power and allow the use of less restrictive prevalence thresholds, thereby enabling the inclusion of low-frequency taxa that may be biologically relevant. In addition, larger sample size would facilitate the application of more robust statistical models tailored to sparse microbiome data, improving the ability to detect subtle differences between endometriosis and control groups. In addition, clinical biomarker data specifically related to endometriosis lesion characteristics were not systematically collected in this study, precluding correlation analyses with microbial profiles. Future studies integrating microbiome data with clinical, biochemical and lesion-specific parameters will be essential to better understand the potential biological and clinical relevance of endometrial microbiota alterations. Despite these limitations, the study also presents notable strengths. Samples were collected during the same menstrual cycle phase (mid-secretory), an important consideration given the cyclical variation of endometrial microbiota (Krog et al., 2022). Furthermore the inclusion of untreated women with active endometriosis avoided the potential confounding effects of hormonal therapy, which has been reported in previous studies (Pérez-Prieto et al., 2024).

## 5 Conclusion

In conclusion, this pilot study supports the existence of viable bacteria in endometrium, but the biomass is low, and the profiles are highly heterogeneous between the individuals. Importantly, the culturomics component of this work should be regarded as a pilot feasibility exercise. While it demonstrates that viable bacteria can be recovered from stored endometrial tissue, the taxonomic profiles might not fully represent the *in vivo* community due to sampling constraints, potential carry-over, and cryopreservation-associated biases. Taken together, these findings support the value of integrating culture-based and sequencing approaches as complementary tools for methodological exploration in low-biomass uterine samples, while highlighting the need for further protocol optimisation and standardisation. Also, the recovery of viable isolates through culturomics in future high-resolution analyses, such as whole-genome sequencing, would provide deeper understanding of the microbe-endometriosis axis. Ultimately, functional analyses of microbial activity and host–microbe interactions, rather than taxonomic profiling alone, will be necessary to clarify the role of the endometrial microbes in reproductive health and disease.

## Funding

This study was funded by the Spanish Ministry of Economy, Industry, and Competitiveness (MINECO) and the European Regional Development Fund (FEDER) through the MCIN/AEI/10.13039/501100011033 and the ERFD’s “A Way of Making Europe” initiative, specifically supporting the Endo-Map (PID2021–12728OB-I00), ROSY (CNS2022–135999), and ENDO-BIOME (PID2024.162334OB.I00) projects. Additionally, S.A. obtained a mobility grant for senior researchers to do research stay abroad funded by the Spanish Ministry of Science, Innovation and Universities (ref. PRX24/00372). C.M.T. is supported by the FPU23/01576 grant, awarded by MCIN/AEI/10.13039/501100011033. Funding for A.C.G. and E.V. was provided through the “Plan de Recuperación, Transformación y Resiliencia” via the “Ayudas para la Recualificación del Sistema Universitario Español” and the “Ayudas Margarita Salas para la Formación de Jóvenes Doctores”, at the Universidad de Murcia and Universidad de Jaén, respectively (ref. UJAR01MS). I.P-P. and A.S.L. received funding from the Fundación Ramón Areces Postdoctoral Fellowships, within the XXXIV and XXXV Calls for the Extension of Studies Abroad in Life and Material Sciences. This work also received support from the Swedish Research Council grant no. 2024-02530, 10.13039/501100000329Novo Nordisk Foundation grant no. NNF24OC0092384, Horizon Europe NESTOR grant no. 101120075, 10.13039/501100005189Estonian Research Council (grant No. IUT34–19, TEM-TA28, PRG1076, PSG1082 and TARISTU24-TK20), and Estonian Ministry of Education and Research (grant No. KOGU-HUMB).

## CRediT authorship contribution statement

A. Canha-Gouveia: Conceptualization, Methodology, Investigation, Data curation, Formal analysis, Writing - original draft, Writing - review and editing. C.M. Tenorio: Methodology, Investigation, Data curation, Formal analysis, Writing - original draft, Writing - review and editing. T. Rööp: Investigation, Writing - review and editing. S. Kõljalg: Investigation, Writing - review and editing. I. Smidt: Investigation, Writing - review and editing. E. Sepp: Investigation, Writing - review and editing. J. Štšepetova: Investigation, Writing - review and editing. M. Saare: Investigation, Writing - review and editing. S. Vela: Methodology, Data curation, Formal analysis, Writing - review and editing. I. Pérez-Prieto: Resources, Investigation, Data curation, Formal analysis, Writing - review and editing. E. Vargas: Resources, Investigation, Data curation. J. Mozas: Resources, Writing - review and editing. A. Clavero: Resources, Writing - review and editing. A. Sola-Leyva: Resources, Investigation, Data curation, Writing - review and editing. A. Salumets: Resources, Writing - review and editing. R. Mändar: Conceptualization, Supervision, Resources, Writing - review and editing. S. Altmäe: Conceptualization, Writing - original draft, Writing - review and editing, Funding acquisition, Project administration, Supervision.

## Declaration of competing interest

The authors declare that they have no known competing financial interests or personal relationships that could have appeared to influence the work reported in this paper.

## Data Availability

The 16S rRNA gene sequencing data are uploaded to SRA database: BioProject ID: PRJNA1299796.

## Appendix Supplementary materials

Image, application 1

Image, application 2

Image, application 3

Image, application 4

Image, application 5

Image, application 6

Image, application 7

Image, application 8

Image, application 9

Image, application 10

Image, application 11

Image, application 12

Image, application 13

Image, application 14

Image, application 15

Image, application 16

Image, application 17

Image, application 18

Image, application 19
