# Supplementary material for: Assessing endometrial microbiota in endometriosis: culturomics and sequencing analysis of receptive-phase tissue
Source: Curr Res Microb Sci. 2026 Apr 1;10:100593. doi: 10.1016/j.crmicr.2026.100593 (PMC13091524; doi:10.1016/j.crmicr.2026.100593)
Supplement: Supplementary file 1 [file mmc1.pdf]

**Table S1.** Summary of all culture media used, references, target species and incubation conditions

| **Medium** | **Supplier** | **Supplement Details** | **Targeted species** | **Incubation conditions** |
|---|---|---|---|---|
| Blood agar | Neogen (NCM0040C, Lot: UK319495) | Citrated sheep blood, Oü Mikrolabor (047/10.10.23) | Aerobic and facultative anaerobic bacteria | 3 days, aerobic incubation, 37 ºC |
| Chocolate agar | Neogen (NCM0040C, Lot: UK319495) | Citrated sheep blood, Oü Mikrolabor (047/10.10.23) | Fastidious bacteria | 3 days, microaerobic incubation, 37 ºC[1] |
| MacConkey agar | Neogen, NCM0174A, Lot: UK319500 | NA | Gram-negative bacteria | 3 days, aerobic incubation, 37 ºC |
| Sabouraud agar | Neogen (NCM0008B, Lot: UK319497) | NA | Yeasts, fungi | 7 days, aerobic incubation, room temperature |
| Fastidious Anaerobe Agar | Neogen (NCM0014B, Lot: UK317040) | Citrated sheep blood, Oü Mikrolabor (018/10.10.2023) | Anaerobic bacteria | 7 days, anaerobic incubation, 37 ºC[2] |
| Schaedler agar | Oxoid (CM0437B, Lot: 3718559) | Citrated sheep blood, Oü Mikrolabor (3718559) | Anaerobic bacteria | 7 days, anaerobic incubation, 37 ºC[2] |
| De Man Rogosa Sharpe Agar | Neogen (NCM0035A, Lot: UK307843) | NA | Lactobacilli | 3–4 days, anaerobic incubation, 37 ºC[2] |
| Gardnerella agar | Becton Dickinson GmbH (Lot: 3726482) | NA | *Gardnerella vaginalis* | 3 days, microaerobic incubation, 37 ºC[1] |

NA: Not applicable; [1] SANYO $CO_2$ incubator, MCO-19AIC(UV) with a gas mixture 10% of $CO_2$; [2] Whitley A35 Anaerobic Workstation; UK with a gas mixture consisting of $CO_2$, $H_2$, $N_2$ (5%; 5%; 90%)
