# Supplementary material for: Assessing endometrial microbiota in endometriosis: culturomics and sequencing analysis of receptive-phase tissue
Source: Curr Res Microb Sci. 2026 Apr 1;10:100593. doi: 10.1016/j.crmicr.2026.100593 (PMC13091524; doi:10.1016/j.crmicr.2026.100593)
Supplement: Supplementary file 2 [file mmc2.pdf]

Table S2. Age and body mass index (BMI) of individual participants in each study group.

ID Patient	Group	Age	BMI
G006	Control	37	23.7
G013	Control	29	22.1
G029	Control	32	23.7
G093	Control	31	22.8
G111	Control	31	22.8
G115	Control	34	30.7
G116	Control	39	20.1
G117	Control	38	21.4
G125	Control	40	28.9
G128	Control	32	31.3
G023	Endometriosis	34	16.2
G041	Endometriosis	33	21.0
G043	Endometriosis	35	28.2
G094	Endometriosis	31	24.8
G099	Endometriosis	27	17.9
G103	Endometriosis	40	29.1
G105	Endometriosis	32	22.3
G108	Endometriosis	39	24.2
G113	Endometriosis	29	22.2
G124 *	Endometriosis	34	21.9

*Excluded from the study for lack of microbial growth.
