# Supplementary material for: Assessing endometrial microbiota in endometriosis: culturomics and sequencing analysis of receptive-phase tissue
Source: Curr Res Microb Sci. 2026 Apr 1;10:100593. doi: 10.1016/j.crmicr.2026.100593 (PMC13091524; doi:10.1016/j.crmicr.2026.100593)
Supplement: Supplementary file 3 [file mmc3.pdf]

Table S3. Clinical characteristics of women with endometriosis, including ovarian involvement, hydrosalpinx, adenomyosis, surgical history, infertility diagnosis and diagnostic method. None of the patients were receiving hormonal treatment at the time of endometrial sampling, except patient G124.

ID Patient	Description
**G023**	Normal ovaries with minimal longitudinal dilation possibly corresponding to a fallopian tube and a 50 mm cystic formation with internal levels suggestive of a functional cyst. Right ovarian cyst 25 mm, left hydrosalpinx.
**G041**	Both fallopian tubes permeable, with filling defects suggestive of endometrial polyps or submucosal fibroids. History of polypectomy. Primary infertility. Endometrioma in right ovary 38 mm.
**G043**	Transvaginal ultrasound shows anteverted uterus with homogeneous endometrium in the 1st phase. Regular myometrium. Retrouterine adnexa with two endometriomas (322 mm and 32 mm) in the right adnexa. Grade IV endometriosis involving the rectovaginal septum. No prior surgery or treatment for endometriosis. Achieved pregnancy via IVF on first attempt.
**G094**	Ovarian endometriosis. T-shaped uterus with possible correction during hysteroscopy. Left ovary with 20 mm endometrioma and 19 mm follicle. Right ovary with 19 mm follicle.
**G099**	Ovarian endometriosis. Persistent endometriosis-like cyst, left side, approximately 45 mm.
**G103**	Bilateral ovarian endometriosis.
**G105**	Bilateral ovarian endometriosis.
**G108**	Endometriotic cystectomy (laterality unspecified).
**G113**	Left ovary: Endometriotic cyst, follicular cysts. Right ovary: Endometriotic cystectomy with epithelium and hemosiderophages consistent with endometriotic cyst.
**G124**	Right ovarian endometrioma of 3-4 cm, adenomyosis. Treated for chronic endometritis.
