# Supplementary material for: Assessing endometrial microbiota in endometriosis: culturomics and sequencing analysis of receptive-phase tissue
Source: Curr Res Microb Sci. 2026 Apr 1;10:100593. doi: 10.1016/j.crmicr.2026.100593 (PMC13091524; doi:10.1016/j.crmicr.2026.100593)
Supplement: Supplementary file 4 [file mmc4.pdf]

**Table S4. Clinical laboratory results from hospital-based diagnostic services.** Histopathological assessment included endometrial dating and evaluation for chronic endometritis using CD138 and MUM1 immunohistochemistry. Microbiological analyses included PCR for genital pathogens (*Chlamydia trachomatis, Neisseria gonorrhoeae, Trichomonas vaginalis, Ureaplasma parvum, U. urealyticum, Mycoplasma genitalium, and M. hominis*) and culture for Lactobacillus spp. and other bacteria. "Negative" in the PCR column indicates absence of pathogens. "No growth" in the culture column indicates absence of bacterial growth. Semi-quantitative culture reports are indicated as few or abundant colonies. "Not available" indicates that no result was obtained (sample insufficient or analysis not performed). Tubal metaplasia refers to benign replacement of endometrial epithelium by tubal-type epithelium, without malignant significance.

| Patient ID | Group | Pathology (histopathology) | PCR for pathogens | Culture results |
|---|---|---|---|---|
| G006 | Control | Early secretory endometrium | Not available | Not available |
| G013 | Control | Unsatisfactory sample (mucoid material with strips of endocervical epithelium) | Negative | No growth |
| G029 | Control | Secretory phase endometrium | Not available | Not available |
| G093 | Control | Secretory endometrium without signs of chronic endometritis | Negative | No growth |
| G111 | Control | Advanced secretory endometrium; minimal stromal inflammation (<5 plasma cells), uncertain significance | Negative | No growth |
| G115 | Control | Secretory endometrium; no signs of chronic endometritis | Negative | No growth |
| G116 | Control | Secretory endometrium; few MUM1- and CD38-positive cells consistent with chronic endometritis | Negative | *Lactobacillus iners* (few colonies) |
| G117 | Control | Secretory endometrium with focal signs of chronic endometritis; IHC: single MUM1-positive cell | Negative | *Lactobacillus crispatus* (few colonies), *Lactobacillus jensenii* (abundant colonies) |
| G125 | Control | Secretory endometrium; foci of tubal metaplasia and reactive changes; no endometritis (CD138/MUM1) | Negative | *Gardnerella vaginalis*, *Enterococcus faecalis* |
| G128 | Control | Secretory endometrium with focal stromal oedema; no plasma cells (CD138/MUM1) | Negative | *Lactobacillus crispatus* (abundant colonies) |
| G023 | Endometriosis | Secretory phase endometrium; no signs of chronic endometritis | Not available | Not available |
| G041 | Endometriosis | Scant secretory endometrium in cervical mucus; no signs of chronic endometritis | Negative | No growth |
| G043 | Endometriosis | Secretory phase endometrium | Negative | No growth |
| G094 | Endometriosis | Secretory endometrium; no signs of chronic endometritis | Negative | *Gardnerella vaginalis* |
| G099 | Endometriosis | Secretory endometrium; no signs of chronic endometritis | Negative | *Lactobacillus crispatus* (few colonies) |
| G103 | Endometriosis | Early secretory endometrium; isolated signs of chronic endometritis (~15 plasma cells) | Negative | *Lactobacillus vaginalis* (few colonies) |
| G105 | Endometriosis | Secretory endometrium; 3–4 MUM1-positive cells in one fragment | Positive for *Neisseria gonorrhoeae* | *Gardnerella vaginalis* |
| G108 | Endometriosis | Secretory endometrium; no signs of chronic endometritis (histology/IHC) | Negative | No growth |
| G113 | Endometriosis | Secretory endometrium with nonspecific stromal changes; very few CD138/MUM1+ cells | Positive for *Ureaplasma urealyticum* | *Lactobacillus jensenii*, *Gardnerella vaginalis* (abundant colonies) |
| G124* | Endometriosis | Secretory endometrium; no evidence of endometritis (CD138-/MUM1-) | Negative | No growth |

*Excluded from the study for lack of microbial growth.
