# Supplementary material for: Assessing endometrial microbiota in endometriosis: culturomics and sequencing analysis of receptive-phase tissue
Source: Curr Res Microb Sci. 2026 Apr 1;10:100593. doi: 10.1016/j.crmicr.2026.100593 (PMC13091524; doi:10.1016/j.crmicr.2026.100593)
Supplement: Supplementary file 5 [file mmc5.pdf]

Table S5. Taxonomic classification of the 40 bacterial species identified in the study by culturomics, Gram-staining profile, oxygen requirements, and identification level. Taxa identified only at the genus level by MALDI-TOF MS (e.g., *Peptoniphilus* sp.) were included based on distinct colony morphology and non-redundant identification.

	Phylum	Class	Order	Family	Genus	Species	Gram	Metabolism
1	*Bacteroidota*	*Bacteroidia*	*Bacteroidales*	*Prevotellaceae*	*Prevotella*	*Prevotella melaninogenica*	Negative	Anaerobic
2	*Bacillota*	*Negativicutes*	*Veillonellales*	*Veillonellaceae*	*Dialister*	*Dialister micraerophilus*	Negative	Anaerobic
3		*Tissierellia*	*Tissierellales*	*Peptoniphilaceae*	*Peptoniphilus*	*Peptoniphilus sp.*	Positive	Anaerobic
4					*Finegoldia*	*Finegoldia magna*	Positive	Anaerobic
5		*Bacilli*	*Bacillales*	*Staphylococcaceae*	*Staphylococcus*	*Staphylococcus warneri*	Positive	Facultative Anaerobic
6						*Staphylococcus hominis*	Positive	Facultative Anaerobic
7						*Staphylococcus haemolyticus*	Positive	Facultative Anaerobic
8						*Staphylococcus epidermidis*	Positive	Facultative Anaerobic
9						*Staphylococcus capitis*	Positive	Facultative Anaerobic
10						*Staphylococcus sp.*	Positive	Facultative Anaerobic
11			*Lactobacillales*	*Enterococcaceae*	*Enterococcus*	*Enterococcus faecalis*	Positive	Facultative Anaerobic
12				*Streptococcaceae*	*Streptococcus*	*Streptococcus sanguinis*	Positive	Facultative Anaerobic
13						*Streptococcus anginosus*	Positive	Facultative Anaerobic (microaerophilic)
14						*Streptococcus oralis*	Positive	Facultative Anaerobic
15				*Lactobacillaceae*	*Lactobacillus*	*Lactobacillus vaginalis*	Positive	Facultative Anaerobic
16						*Lactobacillus jensenii*	Positive	Facultative Anaerobic
17						*Lactobacillus iners*	Positive	Facultative Anaerobic
18						*Lactobacillus gasseri*	Positive	Facultative Anaerobic
19				*Lactobacillaceae*	*Lactobacillus*	*Lactobacillus crispatus*	Positive	Facultative Anaerobic
20	*Actinomycetota*	*Coriobacteriia*	*Coriobacteriales*	*Atopobiaceae*	*Atopobium*	*Atopobium sp.*	Positive	Anaerobic
21					*Fannyhessea*	*Fannyhessea vaginae*	Positive	Anaerobic
22		*Actinomycetes*	*Mycobacteriales*	*Corynebacteriaceae*	*Corynebacterium*	*Corynebacterium amycolatum*	Positive	Aerobic or Facultative Anaerobic
23						*Corynebacterium jeikeium*	Positive	Aerobic
24			*Propionibacteriales*	*Propionibacteriaceae*	*Propionibacterium*	*Propionibacterium sp.*	Positive	Anaerobic (aerotolerant)
25					*Cutibacterium*	*Cutibacterium avidum*	Positive	Anaerobic Aerotolerant

26					*Cutibacterium*	*Cutibacterium acnes*	Positive	Anaerobic Aerotolerant
27					*Gardnerella*	*Gardnerella vaginalis*	Variable	Facultative Anaerobic
28			*Bifidobacteriales*	*Bifidobacteriaceae*		*Bifidobacterium dentium*	Positive	Anaerobic
29					*Bifidobacterium*	*Bifidobacterium breve*	Positive	Anaerobic
30						*Bifidobacterium bifidum*	Positive	Anaerobic
31					*Actinomyces*	*Actinomyces urogenitalis*	Positive	Anaerobic
32					*Schaalia*	*Actinomyces radingae*	Positive	Anaerobic or microaerophilic
33			*Actinomycetales*	*Actinomycetaceae*	*Winkia*	*Winkia neuii*	Positive	Anaerobic or Facultative Anaerobic (strain dependent)
34					*Actinotignum*	*Actinotignum schaalii*	Positive	Facultative Anaerobic
35						*Actinotignum sanguinis*	Positive	Anaerobic
36				*Micrococcaceae*	*Rothia*	*Rothia dentocariosa*	Positive	Facultative Anaerobic
37					*Micrococcus*	*Micrococcus luteus*	Positive	Aerobic
38			*Micrococcales*			*Microbacterium paulum*	Positive	Aerobic
39				*Microbacteriaceae*	*Microbacterium*	*Microbacterium oxydans*	Positive	Aerobic
40						*Microbacterium spp*	Positive	Mostly Aerobic
