# Supplementary material for: Assessing endometrial microbiota in endometriosis: culturomics and sequencing analysis of receptive-phase tissue
Source: Curr Res Microb Sci. 2026 Apr 1;10:100593. doi: 10.1016/j.crmicr.2026.100593 (PMC13091524; doi:10.1016/j.crmicr.2026.100593)
Supplement: Supplementary file 6 [file mmc6.pdf]

Table S6. Cultivable bacterial taxa identified by culturomics in endometrial samples from women with and without endometriosis.

The table lists all species isolated by either direct plating or pre-incubation approaches, indicating detection in 16S environmental controls, oxygen tolerance category, Gram reaction, and percentage of patients in which each taxon was recovered. Asterisks denote taxa detected exclusively after pre-incubation (*), or by both direct plating and pre-incubation (**).

	Endometriosis: 24 bacterias (100% Gram positive, 17%aerobic)			
Taxon (genus/species)	Detected in 16S controls	Oxygen tolerance	Gram	% Patients
*Cutibacterium acnes***	No	Anaerobe (aerotolerant)	Gram-positive	60%
*Lactobacillus crispatus***	Yes	Facultative anaerobe	Gram-positive	50%
Gardnerella vaginalis	No	Facultative anaerobe	Gram-variable	40%
Staphylococcus epidermidis	Yes	Facultative anaerobe	Gram-positive	40%
*Lactobacillus jensenii***	Yes	Facultative anaerobe	Gram-positive	30%
*Lactobacillus gasseri***	Yes	Facultative anaerobe	Gram-positive	20%
*Micrococcus luteus***	No	Aerobe	Gram-positive	20%
*Actinomyces urogenitalis***	Yes	Facultative anaerobe	Gram-positive	10%
Bifidobacterium bifidum	No	Obligate anaerobe	Gram-positive	10%
*Bifidobacterium breve**	No	Obligate anaerobe	Gram-positive	10%
Cutibacterium avidum	No	Anaerobe (aerotolerant)	Gram-positive	10%

	Control: 31 bacterias (94% Gram positive, 6%aerobic)			
Taxon (genus/species)	Detected in 16S controls	Oxygen tolerance	Gram	% Patients
*Lactobacillus crispatus***	Yes	Facultative anaerobe	Gram-positive	50%
Gardnerella vaginalis	No	Facultative anaerobe	Gram-variable	40%
*Cutibacterium acnes***	No	Obligate anaerobe (aerotolerant)	Gram-positive	30%
Lactobacillus iners	Yes	Facultative anaerobe (microaerophile)	Gram-positive	30%
*Staphylococcus epidermidis***	Yes	Facultative anaerobe	Gram-positive	30%
*Lactobacillus jensenii***	Yes	Facultative anaerobe	Gram-positive	20%
*Actinomyces radingae**	Yes	Facultative anaerobe (microaerophile)	Gram-positive	10%
*Actinomyces urogenitalis***	Yes	Anaerobe	Gram-positive	10%
*Actinotignum sanguinis***	No	Facultative anaerobe	Gram-positive	10%
*Actinotignum schaalii***	No	Facultative anaerobe	Gram-positive	10%
Atopobium sp.	No	Obligate anaerobe	Gram-positive	10%

Fannyhessea vaginae	No	Obligate anaerobe	Gram-positive	10%
Lactobacillus iners	Yes	Facultative anaerobe (microaerophilic)	Gram-positive	10%
Lactobacillus vaginalis	Yes	Facultative anaerobe	Gram-positive	10%
*Microbacterium oxydans**	No	Aerobe	Gram-positive	10%
*Microbacterium paulum**	No	Aerobe	Gram-positive	10%
*Microbacterium sp.**	No	Aerobe	Gram-positive	10%
*Peptoniphilus sp.***	Yes	Obligate anaerobe	Gram-positive	10%
Propionibacterium sp.	No	Anaerobe (aerotolerant)	Gram-positive	10%
Staphylococcus haemolyticus	Yes	Facultative anaerobe	Gram-positive	10%
*Staphylococcus hominis***	Yes	Facultative anaerobe	Gram-positive	10%
*Staphylococcus warneri***	Yes	Facultative anaerobe	Gram-positive	10%
*Streptococcus anginosus***	No	Facultative anaerobe (microaerophilic)	Gram-positive	10%
*Streptococcus sanguinis***	No	Facultative anaerobe	Gram-positive	10%

* Only detected using the pre incubation approach

**Detected with both approaches direct plating and pre incubation

*Bifidobacterium dentium***	No	Obligate anaerobe	Gram-positive	10%
*Corynebacterium amycolatum**	No	Aerobe or facultative anaerobe	Gram-positive	10%
*Corynebacterium jeikeium**	No	Aerobe	Gram-positive	10%
*Dialister micraerophilus**	Yes	Obligate anaerobe	Gram-negative	10%
*Enterococcus faecalis***	No	Facultative anaerobe	Gram-positive	10%
Fannyhessea vaginae	No	Obligate anaerobe	Gram-positive	10%
*Finegoldia magna**	Yes	Obligate anaerobe	Gram-positive	10%
*Lactobacillus gasseri***	Yes	Facultative anaerobe	Gram-positive	10%
*Peptoniphilus sp.***	Yes	Obligate anaerobe	Gram-positive	10%
Prevotella melaninogenica	Yes	Obligate anaerobe	Gram-negative	10%
*Propionibacterium sp.**	No	Obligate anaerobe (aerotolerant)	Gram-positive	10%
Rothia dentocariosa	Yes	Facultative anaerobe	Gram-positive	10%
*Staphylococcus capitis**	Yes	Facultative anaerobe	Gram-positive	10%
Staphylococcus haemolyticus	Yes	Facultative anaerobe	Gram-positive	10%
*Staphylococcus hominis***	Yes	Facultative anaerobe	Gram-positive	10%

Staphylococcus sp.	Yes	Facultative anaerobe	Gram-positive	10%
*Streptococcus anginosus***	No	Facultative anaerobe (microaerophile/capnophilic)	Gram-positive	10%
Streptococcus oralis	No	Facultative anaerobe	Gram-positive	10%
*Streptococcus sanguinis***	No	Facultative anaerobe	Gram-positive	10%
*Winkia neuii***	No	Aerobe or facultative anaerobe	Gram-positive	10%
