# Supplementary material for: Assessing endometrial microbiota in endometriosis: culturomics and sequencing analysis of receptive-phase tissue
Source: Curr Res Microb Sci. 2026 Apr 1;10:100593. doi: 10.1016/j.crmicr.2026.100593 (PMC13091524; doi:10.1016/j.crmicr.2026.100593)
Supplement: Supplementary file 8 [file mmc8.pdf]

Table S8. Bacterial taxa isolated by culturomics from endometrial samples of patients of control group: incidence and culture method. Bacterial species isolated from receptive-phase endometrial samples of patients with endometriosis are listed. For each patient (G006–G128), the presence of a given species is indicated by “x” under the corresponding culture method: direct plating (D) or culture after pre-incubation in enrichment broth (Pre). The percentage of patients in whom each species was detected is shown in the rightmost column.

Species	G006		G013		G029		G093		G111		G115		G116		G117		G125		G128		% Patients
	Direct	Pre-inc.	Direct	Pre-inc.	Direct	Pre-inc.	Direct	Pre-inc.	Direct	Pre-inc.	Direct	Pre-inc.	Direct	Pre-inc.	Direct	Pre-inc.	Direct	Pre-inc.	Direct	Pre-inc.	
Prevotella melaninogenica													x								10%
Dialister micraerophilus																		x			10%
Finegoldia magna																		x			10%
Peptoniphilus sp.																	x	x			10%
Staphylococcus warneri																					0
Staphylococcus hominis	x	x																			10%
Staphylococcus haemolyticus	x																				10%
Staphylococcus epidermidis	x		x		x																30%
Staphylococcus capitis				x																	10%
Staphylococcus sp.			x																		10%
Enterococcus faecalis																	x	x			10%
Streptococcus sanguinis	x																				10%
Streptococcus oralis																	x				10%
Streptococcus anginosus																		x			10%
Lactobacillus vaginalis																					0
Lactobacillus jensenii															x	x			x	x	20%
Lactobacillus iners					x				x								x				30%
Lactobacillus gasseri							x														10%
Lactobacillus crispatus	x	x									x		x		x				x		50%
Atopobium sp.													x								10%
Fannyhessea vaginae																	x				10%
Corynebacterium amycolatum																				x	10%
Corynebacterium jeikeium																				x	10%

Propionibacterium sp.																			x		10%
Cutibacterium avidum																					0
Cutibacterium acnes	x	x			x	x									x						30%
Gardnerella vaginalis					x		x						x				x				40%
Bifidobacterium dentium																	x	x			10%
Bifidobacterium breve																					0
Bifidobacterium bifidum																					0
Actinomyces urogenitalis																	x	x			10%
Actinomyces radingae																		x			10%
Winkia neuii																	x	x			10%
Actinotignum schaalii																	x	x			10%
Actinotignum sanguinis																	x	x			10%
Rothia dentocariosa	x																				10%
Micrococcus luteus																					0
Microbacterium paulum																					0
Microbacterium oxydans																					0
Microbacterium spp																					0
