# Supplementary material for: Assessing endometrial microbiota in endometriosis: culturomics and sequencing analysis of receptive-phase tissue
Source: Curr Res Microb Sci. 2026 Apr 1;10:100593. doi: 10.1016/j.crmicr.2026.100593 (PMC13091524; doi:10.1016/j.crmicr.2026.100593)
Supplement: Supplementary file 11 [file mmc11.pdf]

Table S11. Taxa detected in environmental and procedural controls and microDecon decontamination summary.

Environmental and procedural controls were collected in parallel with sample acquisition to assess potential contamination. Controls included sterile swabs from gloves, Tao Brush cutting tools, room air, and blank Copan transport tubes, all preserved in Copan eNAT® medium.

The microDecon algorithm (McKnight et al., 2019) was applied to remove taxa detected in these controls from the 16S rRNA dataset.

- **Section A:** Genera and families that remained in clinical samples after *microDecon* adjustment. Their abundance could not be fully explained by contamination detected in negative controls, so they were included in the adjusted dataset
- **Section B:** Taxa identified as contaminants and completely removed from the dataset.
- **Section C:** Taxa detected exclusively in negative controls and not observed in any clinical samples.

**A. Genera remaining after decontamination**			
**OTU_ID**	**Control (environment)**	**Control (gloves)**	**Control (medium)**
Actinomyces	182	34	48
Enhydrobacter	101	40	0
Escherichia–Shigella	267	151	0
Finegoldia	79	49	0
Fusobacterium	13	101	67
Lactobacillus	0	149	14
Lawsonella	88	111	139
Leptotrichia	19	0	65
Meiothermus	0	124	0
Peptoniphilus	68	168	73
Plastorhodobacter	0	0	160
Porphyromonas	10	53	88
Prevotella	114	274	56
Rothia	153	227	164
Staphylococcus	396	288	306
Thermus	130	84	83
Veillonella	140	51	14
Acetobacteraceae	0	49	0
Comamonadaceae	148	212	266
Corynebacteriaceae	159	206	91
Micrococcaceae	0	44	130
Prevotellaceae	34	0	47

B. Genera identified as contaminants and removed by *microDecon*			
**OTU_ID**	**Control (environment)**	**Control (gloves)**	**Control (medium)**
Acidibacter	42	123	0
Acinetobacter	318	484	196
Alloprevotella	331	77	151
Anaerococcus	135	114	23
Bacillus	228	64	0
Bacteroides	2	68	190
Bifidobacterium	0	166	0
Corynebacterium	407	600	410
Gemella	92	136	126
Lactococcus	76	0	47
Leuconostoc	0	0	6
Listeria	91	104	0
Mycobacterium	89	72	193
Prevotella_7	97	19	75
Pseudomonas	216	86	58
Streptococcus	970	235	441
Turicella	76	0	98
Enterobacterales	188	115	116
Neisseriaceae	0	205	167
Rhodobacteraceae	328	520	465
Sphingomonadaceae	246	271	318

C. Taxa detected only in controls (not in samples)			
**OTU_ID**	**Control (environment)**	**Control (gloves)**	**Control (medium)**
Alcanivorax	28	83	22
Ancalomicrobium	0	0	0
Arcticibacter	40	21	0
Cereibacter	102	0	0
Dorea	0	94	0
Elstera	0	0	93
Floricoccus	0	102	0
Lachnospiraceae_ND3007_group	0	0	0
Moraxellaceae	0	0	0
OM27_clade	0	0	96
Polynucleobacter	0	0	0
Shewanella	0	0	0

Spirosoma	0	0	122
Beijerinckiaceae	58	23	61
Halomonadaceae	0	177	0
