# Supplementary material for: Assessing endometrial microbiota in endometriosis: culturomics and sequencing analysis of receptive-phase tissue
Source: Curr Res Microb Sci. 2026 Apr 1;10:100593. doi: 10.1016/j.crmicr.2026.100593 (PMC13091524; doi:10.1016/j.crmicr.2026.100593)
Supplement: Supplementary file 12 [file mmc12.pdf]

Table S12. Genera and families identified through culturomics, 16S rRNA gene sequencing (filter 0.1%), or both methodologies.

16S rRNA gene sequencing	Both	Culturomics
Aerococcus	*Lactobacillus*	*Cutibacterium*
Akkermansia	*Prevotella*	*Streptococcus*
Alcaligenes	*Gardnerella*	*Bifidobacterium*
Alkalibacterium	*Staphylococcus*	*Fannyhessea*
Blautia	*Corynebacterium*	*Micrococcus*
Campylobacter	*Peptoniphilus*	*Propionibacterium*
Chitinophaga	*Finegoldia*	*Actinotignum*
Chloroplast	*Rothia*	*Dialister*
Conservatibacter	*Atopobium*	*Enterococcus*
Dellaglioa	*Actinomyces*	*Microbacterium*
Enhydrobacter	-	*Schaalia*
Escherichia-Shigella	-	*Winkia*
Facklamia	-	-
Faecalibacterium	-	-
Fastidiosipila	-	-
Fusobacterium	-	-
Georgenia	-	-
Helcococcus	-	-
Isobaculum	-	-
Lawsonella	-	-
Leptotrichia	-	-
Megasphaera	-	-
Meiothermus	-	-
Mesocricetibacter	-	-
Mobiluncus	-	-
Moheibacter	-	-
Mycoplasma	-	-
Niveispirillum	-	-
Oceanicella	-	-
Paenirhodobacter	-	-
Pajaroellobacter	-	-
Pediococcus	-	-
Plastorhodobacter	-	-
Pleomorphomonas	-	-
Porphyromonas	-	-
Pseudoglutamicibacter	-	-
Pseudoxanthomonas	-	-
Rubrobacter	-	-
Tepidiphilus	-	-
Tersicoccus	-	-

Thermus	-	-
Timonella	-	-
Veillonella	-	-
Xanthobacter	-	-
Genus belong to Family Acetobacteraceae	-	-
Genus belong to Family Burkholderiaceae	-	-
Genus belong to Family Caldilineaceae	-	-
Genus belong to Family Comamonadaceae	-	-
Genus belong to Family Dermacoccaceae	-	-
Genus belong to Family Dermatophilaceae	-	-
Genus belong to Family Lactobacillaceae	-	-
Genus belong to Family Lactobacillaceae HT002	-	-
Genus belong to Family Microbacteriaceae	-	-
Genus belong to Family Micrococcaceae	-	-
Genus belong to Family Myxococcaceae P3OB-42	-	-
Genus belong to Family Oscillospiraceae UCG-002	-	-
Genus belong to Family Planococcaceae	-	-
Genus belong to Family Prevotellaceae	-	-
Genus belong to Family Sphingobacteriaceae	-	-
Genus belong to OrderBurkholderiales	-	-
Genus belong to Order Lactobacillales	-	-
