# Supplementary figures and images for: Assessing endometrial microbiota in endometriosis: culturomics and sequencing analysis of receptive-phase tissue
Source: Curr Res Microb Sci. 2026 Apr 1;10:100593. doi: 10.1016/j.crmicr.2026.100593 (PMC13091524; doi:10.1016/j.crmicr.2026.100593)

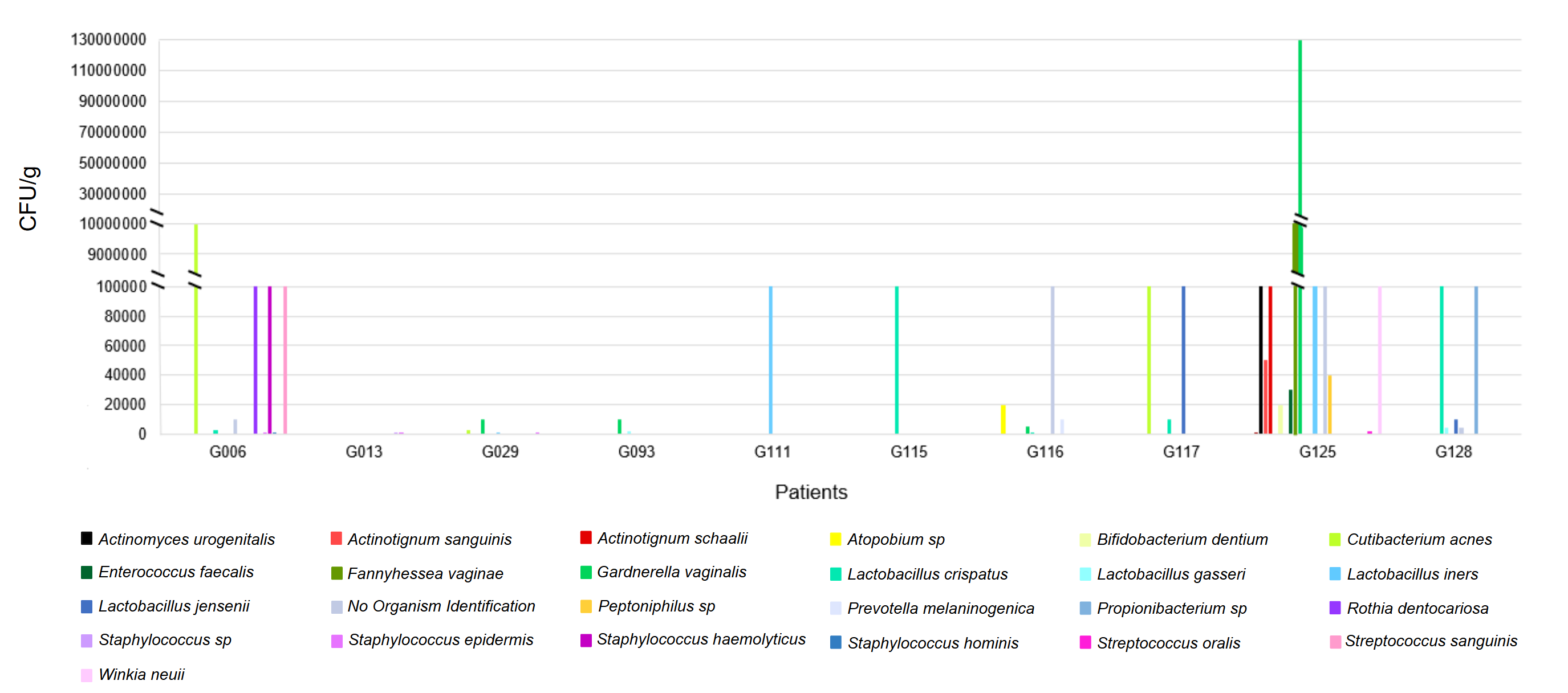

Supplement: Supplementary file 13 [file mmc13.zip › Figure S1.png]

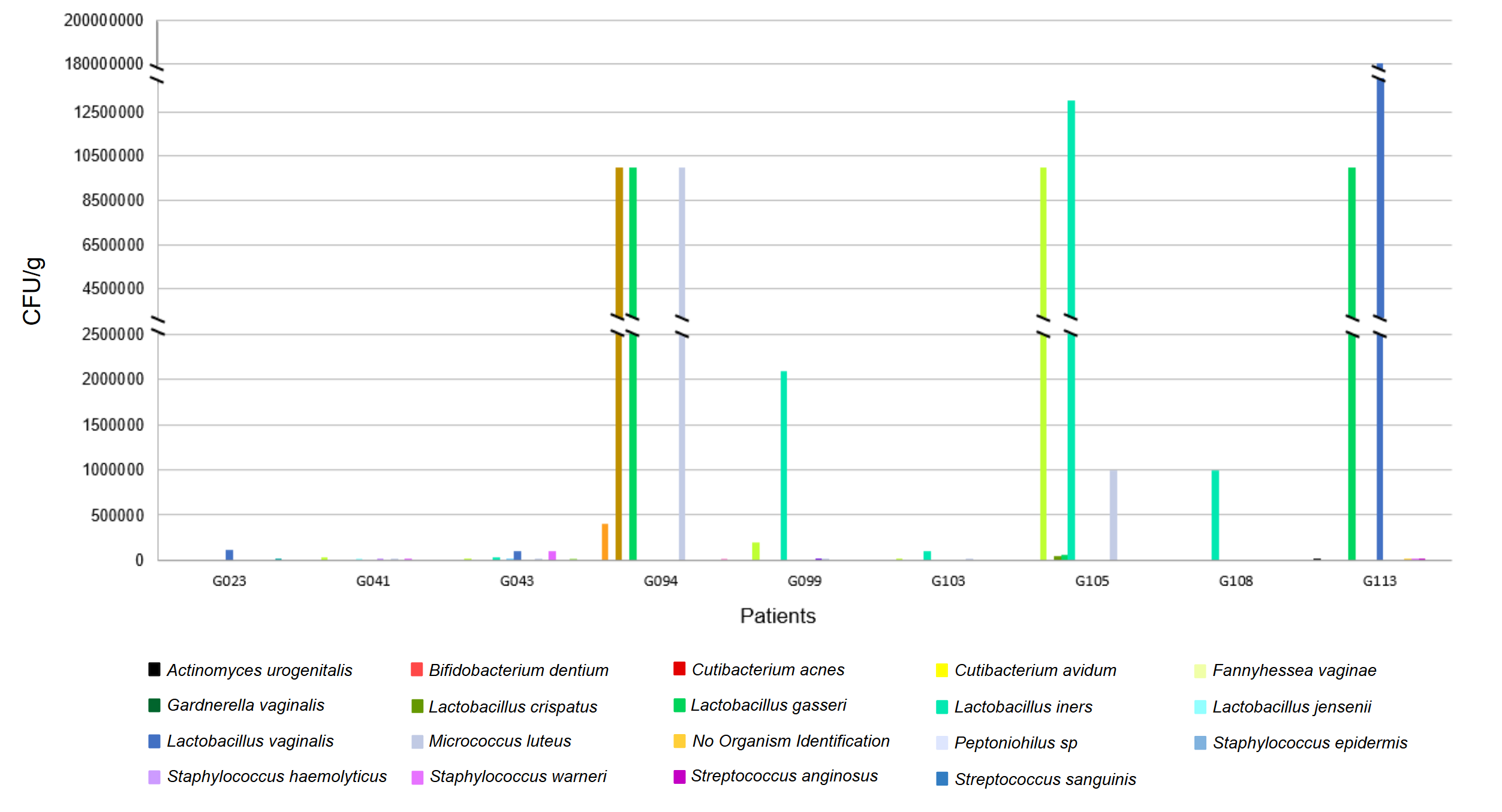

Supplement: Supplementary file 14 [file mmc14.zip › Figure S2.png]

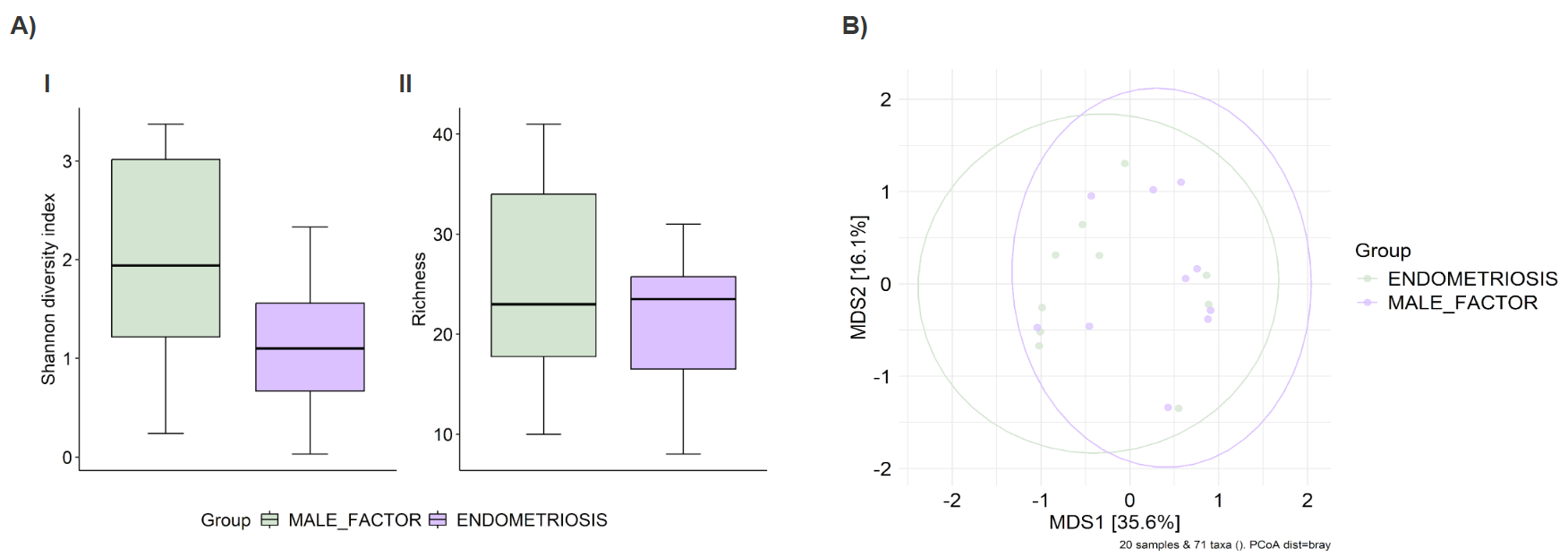

Supplement: Supplementary file 15 [file mmc15.zip › Figure S3.png]

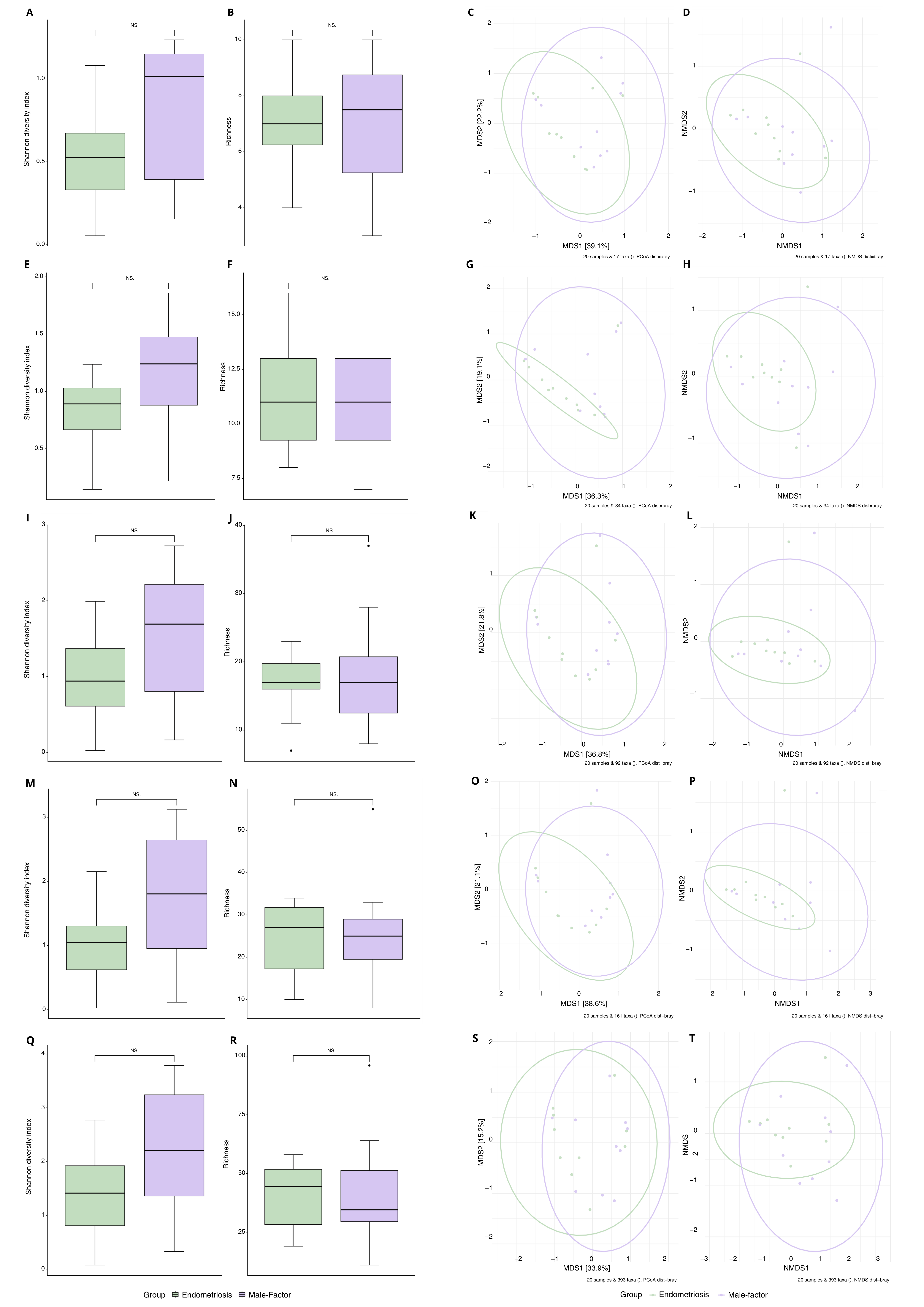

Supplement: Supplementary file 16 [file mmc16.zip › Figure S4.png]

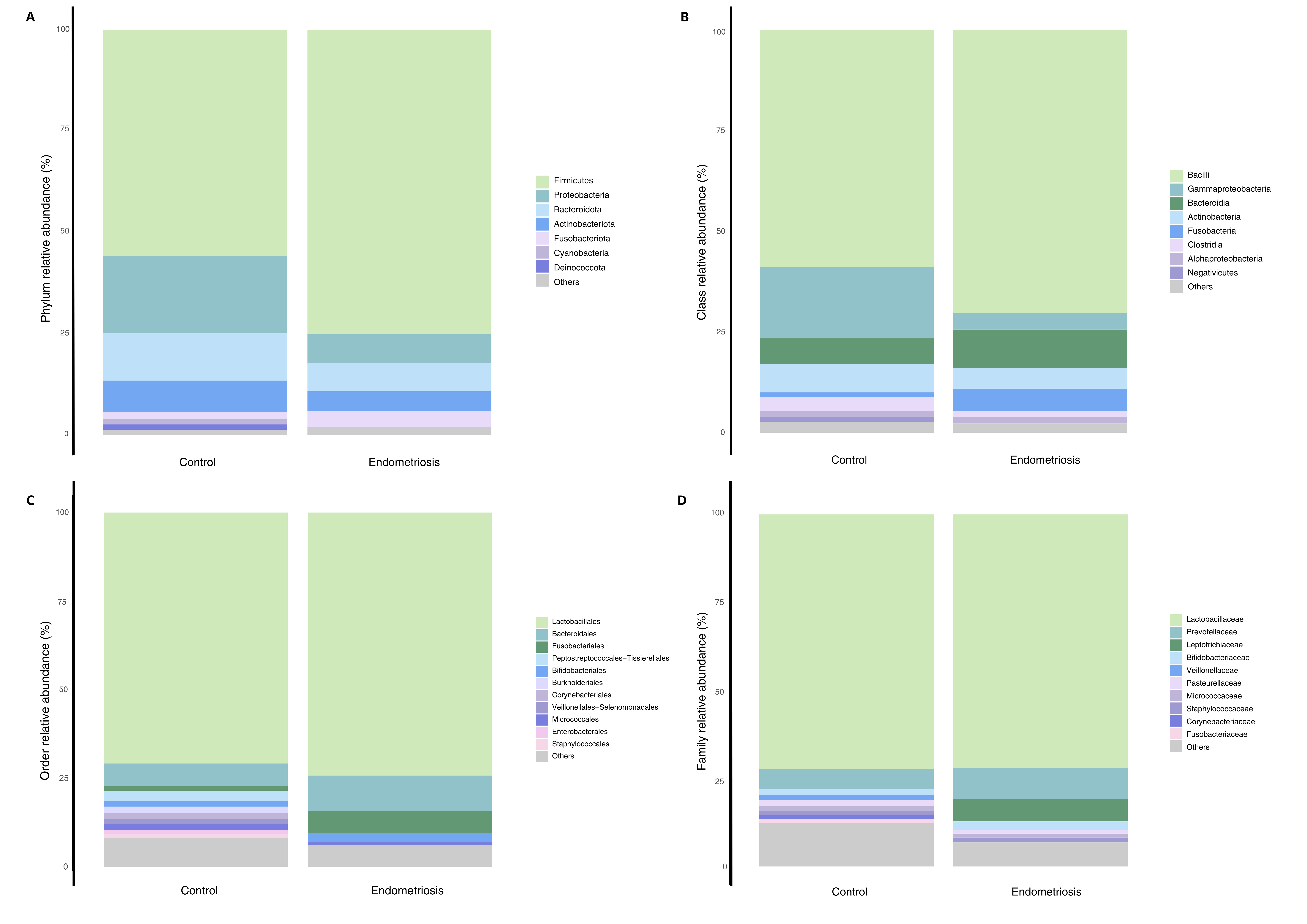

Supplement: Supplementary file 17 [file mmc17.zip › Figure S5.png]
