# Supplementary material for: Assessing endometrial microbiota in endometriosis: culturomics and sequencing analysis of receptive-phase tissue
Source: Curr Res Microb Sci. 2026 Apr 1;10:100593. doi: 10.1016/j.crmicr.2026.100593 (PMC13091524; doi:10.1016/j.crmicr.2026.100593)
Supplement: Supplementary file 9 [file mmc9.pdf]

Table S9. Bacterial taxa isolated by direct culturomics from endometrial samples of patients of control group: quantitative load (log_{10}CFU/g). Bacterial species isolated from receptive-phase endometrial samples of patients without endometriosis are listed with their quantitative counts (log_{10}) colony-forming units per gram of tissue, CFU/g) obtained by direct plating. Patient identifiers range from G006 to G128. Empty cells indicate that the species was not isolated from the corresponding patient under direct culture conditions.

**Species**	**G006**	**G013**	**G029**	**G093**	**G111**	**G115**	**G116**	**G117**	**G125**	**G128**
Lactobacillus crispatus	3.5	0	0	0	0	5	3	4	0	6
Gardnerella vaginalis	0	0	4	4	0	0	5.04	0	8.1	0
Cutibacterium acnes	7	0	3.5	0	0	0	0	6	0	0
Lactobacillus iners	0	0	2	0	5.5	0	0	0	5.5	0
Lactobacillus jensenii	0	0	0	0	0	0	0	5.36	0	4
Staphylococcus epidermidis	2	2	3	0	0	0	0	0	0	0
Fannyhessea vaginae	0	0	0	0	0	0	0	0	7	0
Lactobacillus gasseri	0	0	0	3.3	0	0	0	0	0	3.6
Actinomyces urogenitalis	0	0	0	0	0	0	0	0	5.6	0
Staphylococcus haemolyticus	5	0	0	0	0	0	0	0	0	0
Streptococcus sanguinis	5	0	0	0	0	0	0	0	0	0
Propionibacterium sp.	0	0	0	0	0	0	0	0	0	5
Winkia neuii	0	0	0	0	0	0	0	0	5	0
Actinotignum schaalii	0	0	0	0	0	0	0	0	5	0
Rothia dentocariosa	5	0	0	0	0	0	0	0	0	0
Actinotignum sanguinis	0	0	0	0	0	0	0	0	4.7	0
Peptoniphilus sp.	0	0	0	0	0	0	0	0	4.6	0
Enterococcus faecalis	0	0	0	0	0	0	0	0	4.5	0
Atopobium sp.	0	0	0	0	0	0	4.3	0	0	0
Bifidobacterium dentium	0	0	0	0	0	0	0	0	4.3	0
Prevotella melaninogenica	0	0	0	0	0	0	4	0	0	0
Streptococcus oralis	0	0	0	0	0	0	0	0	3.3	0
Staphylococcus hominis	3	0	0	0	0	0	0	0	0	0
Staphylococcus sp.	0	2	0	0	0	0	0	0	0	0
Cutibacterium avidum	0	0	0	0	0	0	0	0	0	0
Bifidobacterium bifidum	0	0	0	0	0	0	0	0	0	0
Staphylococcus warneri	0	0	0	0	0	0	0	0	0	0
Lactobacillus vaginalis	0	0	0	0	0	0	0	0	0	0
Micrococcus luteus	0	0	0	0	0	0	0	0	0	0
Streptococcus anginosus	0	0	0	0	0	0	0	0	0	0
Dialister micraerophilus	0	0	0	0	0	0	0	0	0	0
Finegoldia magna	0	0	0	0	0	0	0	0	0	0
Staphylococcus capitis	0	0	0	0	0	0	0	0	0	0
Corynebacterium amycolatum	0	0	0	0	0	0	0	0	0	0
Corynebacterium jeikeium	0	0	0	0	0	0	0	0	0	0
Bifidobacterium breve	0	0	0	0	0	0	0	0	0	0
Actinomyces radingae	0	0	0	0	0	0	0	0	0	0
Microbacterium paulum	0	0	0	0	0	0	0	0	0	0
Microbacterium oxydans	0	0	0	0	0	0	0	0	0	0
Microbacterium spp	0	0	0	0	0	0	0	0	0	0
