# Supplementary material for: Assessing endometrial microbiota in endometriosis: culturomics and sequencing analysis of receptive-phase tissue
Source: Curr Res Microb Sci. 2026 Apr 1;10:100593. doi: 10.1016/j.crmicr.2026.100593 (PMC13091524; doi:10.1016/j.crmicr.2026.100593)
Supplement: Supplementary file 10 [file mmc10.pdf]

Table S10. Bacterial taxa isolated by direct culturomics from endometrial samples of patients with endometriosis: quantitative load (log10CFU/g). Bacterial species isolated from receptive-phase endometrial samples of patients with endometriosis are listed with their quantitative counts (log10) colony-forming units per gram of tissue, CFU/g) obtained by direct plating. Patient identifiers range from G023 to G124. Empty cells indicate that the species was not isolated from the corresponding patient under direct culture conditions.

Species	G023	G041	G043	G094	G099	G103	G105	G108	G113	G124
Lactobacillus crispatus	0	0	4.5	0	6.3	5	7.1	6	0	0
Cutibacterium acnes	0	4.5	4	0	5.3	3	7	0	0	0
Gardnerella vaginalis	0	0	0	7	0	0	4.8	0	7	0
Lactobacillus jensenii	5.1	0	5	0	0	0	0	0	8.3	0
Staphylococcus epidermidis	0	3	5	0	0	0	0	0	3	0
Cutibacterium avidum	0	0	0	7	0	0	0	0	0	0
Bifidobacterium bifidum	0	0	0	5.6	0	0	0	0	0	0
Fannyhessea vaginae	0	0	0	0	0	0	4.7	0	0	0
Lactobacillus iners	0	0	3.6	0	0	0	0	0	0	0
Lactobacillus gasseri	0	3.3	0	0	0	0	0	0	0	0
Staphylococcus haemolyticus	0	0	0	0	0	0	0	0	3	0
Streptococcus sanguinis	0	0	0	3	0	0	0	0	0	0
Staphylococcus warneri	3	0	0	0	0	0	0	0	0	0
Lactobacillus vaginalis	0	3	0	0	0	0	0	0	0	0
Micrococcus luteus	0	0	0	0	3	0	0	0	0	0
Actinomyces urogenitalis	0	0	0	0	0	0	0	0	2.3	0
Peptoniphilus sp.	0	0	0	0	0	0	0	0	2.3	0
Streptococcus anginosus	0	0	2	0	0	0	0	0	0	0
Propionibacterium sp.	0	0	0	0	0	0	0	0	0	0
Winkia neuii	0	0	0	0	0	0	0	0	0	0
Actinotignum schaalii	0	0	0	0	0	0	0	0	0	0
Rothia dentocariosa	0	0	0	0	0	0	0	0	0	0
Actinotignum sanguinis	0	0	0	0	0	0	0	0	0	0
Enterococcus faecalis	0	0	0	0	0	0	0	0	0	0
Atopobium sp.	0	0	0	0	0	0	0	0	0	0
Bifidobacterium dentium	0	0	0	0	0	0	0	0	0	0
Prevotella melaninogenica	0	0	0	0	0	0	0	0	0	0
Streptococcus oralis	0	0	0	0	0	0	0	0	0	0
Staphylococcus hominis	0	0	0	0	0	0	0	0	0	0
Staphylococcus sp.	0	0	0	0	0	0	0	0	0	0
Dialister micraerophilus	0	0	0	0	0	0	0	0	0	0
Finegoldia magna	0	0	0	0	0	0	0	0	0	0
Staphylococcus capitis	0	0	0	0	0	0	0	0	0	0
Corynebacterium amycolatum	0	0	0	0	0	0	0	0	0	0
Corynebacterium jeikeium	0	0	0	0	0	0	0	0	0	0
Bifidobacterium breve	0	0	0	0	0	0	0	0	0	0
Actinomyces radingae	0	0	0	0	0	0	0	0	0	0
Microbacterium paulum	0	0	0	0	0	0	0	0	0	0
Microbacterium oxydans	0	0	0	0	0	0	0	0	0	0
Microbacterium spp	0	0	0	0	0	0	0	0	0	0
